# High-resolution transcriptomics informs glial pathology in human temporal lobe epilepsy

**DOI:** 10.1186/s40478-022-01453-1

**Published:** 2022-10-23

**Authors:** Balagopal Pai, Jessica Tome-Garcia, Wan Sze Cheng, German Nudelman, Kristin G. Beaumont, Saadi Ghatan, Fedor Panov, Elodia Caballero, Kwadwo Sarpong, Lara Marcuse, Jiyeoun Yoo, Yan Jiang, Anne Schaefer, Schahram Akbarian, Robert Sebra, Dalila Pinto, Elena Zaslavsky, Nadejda M. Tsankova

**Affiliations:** 1 Department of Pathology and Laboratory Medicine, Icahn School of Medicine at Mount Sinai, New York, NY 10029 USA; 2 Department of Neuroscience and Friedman Brain Institute, Icahn School of Medicine at Mount Sinai, New York, NY 10029 USA; 3 Department of Neurology, Icahn School of Medicine at Mount Sinai, New York, NY 10029 USA; 4 Department of Genetics & Genomic Sciences, Icahn School of Medicine at Mount Sinai, New York, NY 10029 USA; 5 Icahn Institute for Data Science and Genomic Technology, New York, NY 10029 USA; 6 Department of Neurosurgery, Icahn School of Medicine at Mount Sinai, New York, NY 10029 USA; 7 Department of Psychiatry, Icahn School of Medicine at Mount Sinai, New York, NY 10029 USA

## Abstract

The pathophysiology of epilepsy underlies a complex network dysfunction between neurons and glia, the molecular cell type-specific contributions of which remain poorly defined in the human disease. In this study, we validated a method that simultaneously isolates neuronal (NEUN +), astrocyte (PAX6 + NEUN–), and oligodendroglial progenitor (OPC) (OLIG2 + NEUN–) enriched nuclei populations from non-diseased, fresh-frozen human neocortex and then applied it to characterize the distinct transcriptomes of such populations isolated from electrode-mapped temporal lobe epilepsy (TLE) surgical samples. Nuclear RNA-seq confirmed cell type specificity and informed both common and distinct pathways associated with TLE in astrocytes, OPCs, and neurons. Compared to postmortem control, the transcriptome of epilepsy astrocytes showed downregulation of mature astrocyte functions and upregulation of development-related genes. To gain further insight into glial heterogeneity in TLE, we performed single cell transcriptomics (scRNA-seq) on four additional human TLE samples. Analysis of the integrated TLE dataset uncovered a prominent subpopulation of glia that express a hybrid signature of both reactive astrocyte and OPC markers, including many cells with a mixed *GFAP* + *OLIG2* + phenotype. A further integrated analysis of this TLE scRNA-seq dataset and a previously published normal human temporal lobe scRNA-seq dataset confirmed the unique presence of hybrid glia only in TLE. Pseudotime analysis revealed cell transition trajectories stemming from this hybrid population towards both OPCs and reactive astrocytes. Immunofluorescence studies in human TLE samples confirmed the rare presence of GFAP + OLIG2 + glia, including some cells with proliferative activity, and functional analysis of cells isolated directly from these samples disclosed abnormal neurosphere formation in vitro. Overall, cell type-specific isolation of glia from surgical epilepsy samples combined with transcriptomic analyses uncovered abnormal glial subpopulations with de-differentiated phenotype, motivating further studies into the dysfunctional role of reactive glia in temporal lobe epilepsy.

**Supplementary Information:**

The online version contains supplementary material available at 10.1186/s40478-022-01453-1.

## Introduction

Epilepsy is a debilitating neurological disorder that affects ~ 0.5–1% of the population [1]. The disease has been predominantly studied in the context of neuronal excitability and network dysfunction; yet, therapeutic reduction of neuronal activity has shown only limited clinical efficacy [2, 3]. More recently, pathogenic roles for glia and neuroinflammation have emerged, implicating a more complex but intimate dysregulation of the glial-neuronal homeostasis in epilepsy [4, 6]. Rodent studies have begun to elucidate the cell type-specific molecular pathways dysregulated in epilepsy [5]. Oligodendroglial progenitors (OPCs), also referred to as NG2-positive glia, known to proliferate at a low level under physiological conditions, have been shown to increase in number and migrate to the site of brain injury under various central nervous system (CNS) insults [7–12], including epileptic activity [13], where their presence has been implicated in subtle myelin dysregulation [14, 15]. In contrast, astrocytes remain within their niche, where they can alter their phenotype in response to injury [16], including in the context of seizures [17, 18]. At the cellular level, reactive astrocytes in epileptic lesions show dysregulation of potassium (K +) channels, glutamate transporters, aquaporins, and connexins [6, 19, 20].

Characterizing the functional and molecular biology of glia in *human* TLE pathology, however, has been more limited, in part due to the difficulty of dissociating glia and neurons in primary tissue, the cytoplasmic processes of which are heavily interconnected [21]. Fluorescence-activated nuclei sorting (FANS) has emerged as a powerful tool to isolate and study human neuronal nuclei (NEUN +) populations from fresh-frozen archival tissue [22, 23], circumventing cytoplasmic dissociation and minimizing transcriptional activation during processing, and several recent studies have successfully profiled the full transcriptome and open chromatin landscape of neuronal NEUN + populations in both healthy and diseases conditions [24–26]. However, similar methods to isolate specific glial subpopulations from the non-neuronal (NEUN–) element (composed of endothelium, pericytes, smooth muscle cells, inflammatory cells, and all glial subtypes) are lacking in the field and therefore much less is understood about the specific molecular alterations of glial subtypes within the diseased tissue niche [21, 22, 27].

Here, we developed a strategy that uses three transcription factors, NEUN, OLIG2, and PAX6, to simultaneously isolate neuronal, OPC, and astrocyte nuclei populations from non-diseased fresh-frozen postmortem human brain tissue and validated its cell-type specificity using bulk RNA-seq transcriptomics. We then employed it, in combination with single cell RNA-seq, to characterize the cell type-specific transcriptome alterations in primary TLE neocortex.

## Methods

### Sample collection

All tissue samples were obtained de-identified under approved Institutional Review Board (IRB) protocols and appropriate consent. Tissue was either fresh-frozen for FANS, fixed in 4% paraformaldehyde for immunofluorescence (IF) studies, or collected fresh in live cell medium (PIPES) for single cell dissociation. Epilepsy tissue was obtained within 5–30 min of surgical resection, from patients with medically refractory TLE with recent depth electrode recording of primary epileptic activity. For FANS and IF, control tissue was obtained from autopsy adult temporal lobe neocortex (TL) or pediatric germinal matrix with post-mortem interval less than 24 h and without diagnostic neuropathological abnormalities.

### Fluorescence Activated Nuclei Sorting (FANS)

We modified the existent FANS protocol for isolation of NEUN + nuclei from fresh-frozen human brain cortex [22, 23] by including positive selection for astrocyte (PAX6 + or SOX9 +) and OPC (OLIG2 +) enriched populations. Briefly, 200-500 mg of frozen tissue was first manually homogenized using dounce glass grinders (Wheaton; 50 strokes) in a hypotonic lysis buffer (0.32 M Sucrose/5 mM CaCl_2_/3 mM Mg(Ac)_2_/0.1 mM EDTA, 10 mM Tris–HCL pH8/1 mM DTT/0.1% Triton X-100) (PMID: 33938880). Nuclei were collected in this buffer and purified from cellular debris by ultracentrifugation (107,163.6 × g for 1 h at 4 °C) in a sucrose gradient (61.8%) (1.8 M Sucrose/3 mM Mg(Ac)_2_/1 mM DTT/10 mM Tris–HCL pH8). The nuclei pellet was resuspended in 1X PBS, and then nuclei were simultaneously incubated with the three fluorescently-conjugated primary antibodies (0.1% BSA/1X PBS) for one hour at 4 °C on rotation: mouse anti-NEUN-AF555 (Millipore, MAB377A5, 1:1000); mouse anti-PAX6-APC (Novus Biologicals, NBP2-34705APC, 1:1000) or mouse anti-SOX9-AF647 (BD, 565,493, 1:1000); and mouse anti-OLIG2-AF488 (Millipore, MABN50A4, 1:1000). DAPI (1:1000) was added after primary antibody incubation and before FANS (FACSAria™ III sorter; BD Biosciences). Nuclei were collected in Trizol LS (Life Technologies; 3:1 Trizol:nuclei ratio; for up to 50 K nuclei) or in regular Trizol (Life Technologies; 750μL; for more than 50 K nuclei), after first concentrating the solution in 1XPBS containing 0.36 M Sucrose, 3.6 mM Mg(Ac)_2_, 2 mM Tris–HCL pH8, 5 mM CaCl_2_, and were snap-frozen at -80 °C for subsequent RNA isolation.

### Bulk RNA-seq preparation and analysis

Total nuclear RNA was isolated from FANS populations (NEUN + , NEUN–OLIG2 + and NEUN–PAX6 +) by standard phenol /chloroform extraction, followed by DNase digestion (15 min), and RNA cleanup and concentration in final volume of 15µL water (Zymo Research, R1013). RNA concentration was determined using Qubit (ThermoFisher). For FANS RNA-seq library preparation (SMARTer Stranded Total RNA-Seq Kit Pico Input Mammalian, Clontech Laboratories, 635005), 2952 pg of total nuclear RNA were amplified into cDNA with fragmentation times of 2.5 min for autopsy and 3.5 min for TLE cases (14 cycles total amplification for all). Ribosomal RNA depletion was performed using human-specific R-Probes. Libraries were generated using Nextera XT (FC-131–1024) and validated using Agilent 2100 Bioanalyzer. Sequencing was performed on Illumina HiSeq 2500 (50 bp pair-end sequencing, 38–50 million paired-end reads/sample).

Sequenced output FASTQ files of bulk RNA-seq data were assessed for quality using the FASTQC package. Reads were aligned to the human genome (GENCODE GRCh38) using STAR with default settings [28]. Gene counts were obtained using the featureCount utility [29]. The counts data were rld (rlog transformed counts)-normalized. Differential expression analysis was performed using the DESeq2 R package [30], modeling the data with a negative binomial distribution and using Empirical Bayes shrinkage for dispersion and fold change estimation. Functional and gene set enrichment analyses were performed using several tools: HOMER [31] with background defined as the set of genes passing the independent filtering low expression threshold by DESeq2; DAVID [32], and GSEA [33].

### RT-qPCR analysis

FANS RNA was also used to generate cDNA for RT-qPCR (High-Capacity RNA-to-cDNA Kit, Life Technologies, 4387406). Real time PCR was run in duplicates using the SYBR-Green system (Quanta Biosciences, 101414) (7900HT, Life Technologies). Primers spanned exon-exon junctions; melting curves were analyzed to ensure primers specificity; genomic DNA was used as negative control. ACTB was used as housekeeping gene, HKG. Fold gene expression was calculated as 2^–(CtSample – CtHKG)^ relative to the NEUN + fraction (2^−ΔΔCt^).

### Droplet-based single cell RNA-seq preparation and analysis

TLE tissue from five different de-identified patients was obtained from the operating room and immersed in freshly prepared live cell buffer (PIPES) for single cell dissociation. Single cell RNA-seq was performed on these samples using the Chromium platform (10 × Genomics, Pleasanton, CA) with the 3’ gene expression (3’ GEX) V1 kit for 2 samples, V2 kit for 2 samples, and V3 for 1 sample, using an input of ~ 10,000 single cells. Briefly, Gel-Bead in Emulsions (GEMs) were generated on the sample chip in the Chromium controller. Barcoded cDNA was extracted from the GEMs by Post-GEM RT-cleanup and amplified for 12 cycles. Amplified cDNA was fragmented and subjected to end-repair, poly A-tailing, adapter ligation, and 10X-specific sample indexing following the manufacturer’s protocol. Libraries were quantified using Bioanalyzer (Agilent) and Qubit (Thermo Fisher) analysis. Libraries from 1000–4000 cells (depending on the sample) were sequenced in paired-end mode on a HiSeq 4000 instrument (Illumina, San Diego, CA).

#### Primary Sequence Analysis

Cell Ranger v6.1.1 package [34] was used to demultiplex cellular barcodes, align the reads to hg38 reference genome, filter reads outside of cells and count unique molecular identifiers (UMI) per gene, producing a feature-barcode matrix per sample. The filtered feature-barcode matrix from each sample was processed with Seurat v4.1 [35] and normalized using the default “LogNormalize” option and scale.factor = 10,000. Poor quality cells (mitochondrial content of over 20%, or feature counts less than 200, or percent of single-cell dissociation-affected gene expression over 9% [36]), were filtered out. Next, doublets, called as cells with feature counts over 4500, were removed. Finally, rare cells undergoing cell cycle transition (S or G2M phase cell-cycle score greater than 0.15) were filtered out as well due to clustering by state rather than lineage. Based on low-quality metrics (median UMI counts per cells < 200), sample 12814 was removed from subsequent downstream computational analyses. The Seurat package was also used for data integration and scaling, based on the common features among the top 2000 variable features from each of the remaining four samples (13059, 14431, 19619, and 20188). Next, principal component analysis (PCA) and clustering were performed for downstream analysis. UMAP dimensionality reduction was applied for visualization.

#### Cell Type Identification

The SingleR package (v1.6.1) [37] was used to perform an initial cell-type annotation using a reference dataset from the Allen Brain Atlas [38]. Cell type annotations were further validated by (1) canonical marker expression in each annotated cell type and (2) examining differentially expressed genes among the clusters.

#### Normal TL data integration

 For integrated comparison of our scRNA-seq TLE dataset to normal TL control, we used a previously published scRNA-seq TL dataset [39]. Raw data from [39] was downloaded from the Sequence Read Archive (SRA), mapped to the hg38 genome version using STAR (2.7.9). Sequencing QC was ascertained using the fastqc package. “FeatureCounts” from Subread package (2.0.3) was used to generate a counts matrix. Only cells annotated with high confidence by the original authors were used, and derived from temporal cortex, except for OPCs, for which we included cortex and hippocampus to increase total yield. Normal TL data was integrated with TLE data based on common features among the top 2000 variable features as described above.

#### Modulescore and Trajectory Analysis

Top 50 differentially expressed markers of Astrocytes, OPCs, Oligodendrocytes and Microglia from normal TL data [39] were used to calculate signature enrichment scores using the “AddModulescore” function of Seurat. To assess module score differences between cell type clusters, unpaired Wilcoxon rank test was performed. Multiple testing correction of p-values was done using the Benjamini–Hochberg method. Single-cell trajectory and pseudotime analysis was done using the Monocle3 package [40]. Cells were clustered and pseudo temporally ordered, and trajectories visualized over UMAPs.

### In vitro proliferation assay

To assess proliferation, EGFR + (CD34–CD45–) and EGFR– (CD34–CD45–) cells were isolated from ~ 500 mg of fresh human TLE tissue as previously described [41, 42], and were seeded immediately after FACS on 96-well low-adherence plates at a density of 10c/μl, in triplicates, in NS media (1X N2, 1X B27, 20 μM glutamine, 1X Insulin/Transferrin/Selenium, 15 mM HEPES, 0.6% glucose, 1X Antibiotic/Antimycotic, in DMEM/F12 media) supplemented with EGF (20 ng/ml) and bFGF (20 ng/ml). Cells were maintained at 37 °C and 5% CO2 changing 2/3 of media on day 6 and every 3–4 days thereafter. Images of NS formation were captured with a light inverted microscope (Motic AE31) 2 weeks after seeding. Pictures covering the entire surface of the wells were taken at 10X and were used for subsequent counting.

### Immunofluorescence

Specimens were fixed in 4% paraformaldehyde/1X PBS at 4 °C for 24 h (TL/TLE cases), and up to 72 h in the case of germinal matrix, rinsed in 1X PBS, and vibratome sectioned (30 µm). Sections were incubated for 1 h in blocking solution (1X PBS/0.5% Triton X-100/10% normal donkey serum); then for 24 h at 4 °C in primary antibody (1X PBS/0.25% Triton X-100/1% normal donkey serum); and then for 4 h at room temperature in either donkey anti-mouse, donkey anti-rabbit, donkey anti-rat, or donkey anti-goat fluorochrome-conjugated secondary antibodies (Jackson Laboratories, 1:250 dilution). Formalin-fixed paraffin embedded (FFPE) tissues underwent 1 h deparaffinization, rehydration in decreasing gradient of ethanol, and antigen retrieval for 20 min prior to blocking. Primary antibodies dilutions were as follows: 1:50 mouse anti-EGFR (Invitrogen 280005); 1:500 rat anti-GFAP (Life Technologies, 13–0300); 1:250 rabbit anti-OLIG2 (Millipore, AB9610); 1:250 goat anti-hOLIG2 (R&D Systems, AF2418SP); 1:250 rabbit anti-Ki67 (Abcam, Ab15580); 1:250 mouse anti-Ki67 (BD Biosciences, 556003); 1:100 rabbit anti-PAX6 (Novus Biologicals, NBP1-89100); 1:100 mouse anti- NEUN (Millipore, MAB377); 1:250 rabbit anti-AIF1 (IBA1) (Wako, 019–19741). Nuclei were counterstained with DAPI (1:1000). Images were obtained using a LSM 780 upright confocal microscope (Zeiss).

### Statistical analysis

Two-tailed and one-tailed unpaired Student’s t-test was used to calculate significance (*p < 0.05, **p < 0.01, ***p < 0.001), assuming homogeneous variances. For non-parametric analysis, we used the Mann–Whitney U test. Bar graph data is represented as mean ± SEM of at least three independent experiments. All RNA-seq tests were FDR adjusted for multiple testing correction. To assess module score differences between cell type clusters, unpaired Wilcoxon rank test was performed. Multiple testing correction of p-values was done using the Benjamini–Hochberg method.

## RESULTS

### Simultaneous isolation of astrocyte, neuronal, and OPC-enriched nuclei from bulk fresh-frozen human cortex

The role of human glia in many neurological disorders is still poorly understood due to the lack of tools that reliably isolate specific glial subpopulations from bulk tissue, directly from their native niche. To better understand the contributions of glial pathology in human epilepsy, we sought to develop a method that isolates astrocyte and oligodendroglial-lineage populations, two functionally distinct glial cell types, directly from human brain tissue. To do this, we modified the FANS NEUN + /– method for isolating neuronal nuclei from fresh-frozen cortex [22, 23] by incorporating positive selection nuclear markers for astrocyte and OPC nuclei. For OPC isolation, we used OLIG2, a known marker of adult oligodendroglial lineage cells, which shows stronger expression in OPCs compared to mature oligodendrocytes [43–45]. To find a suitable nuclear astrocytic marker, we searched for astrocyte-enriched transcription factor (TF) genes within the HepaCAM-purified resting human astrocyte transcriptome database [46] and found *PAX6* and *SOX9* to be among the top upregulated astrocyte-specific genes. Although largely studied in the context of early neuroepithelial development and retinal neuronal specification [47–49], PAX6 has been shown to promote the maturation of murine astrocytes [50] as well as to co-express with the astrocytic marker GFAP in epileptic human tissue [51], and SOX9 is widely expressed by mouse and human astrocytes [27].

**Fig. 1:**
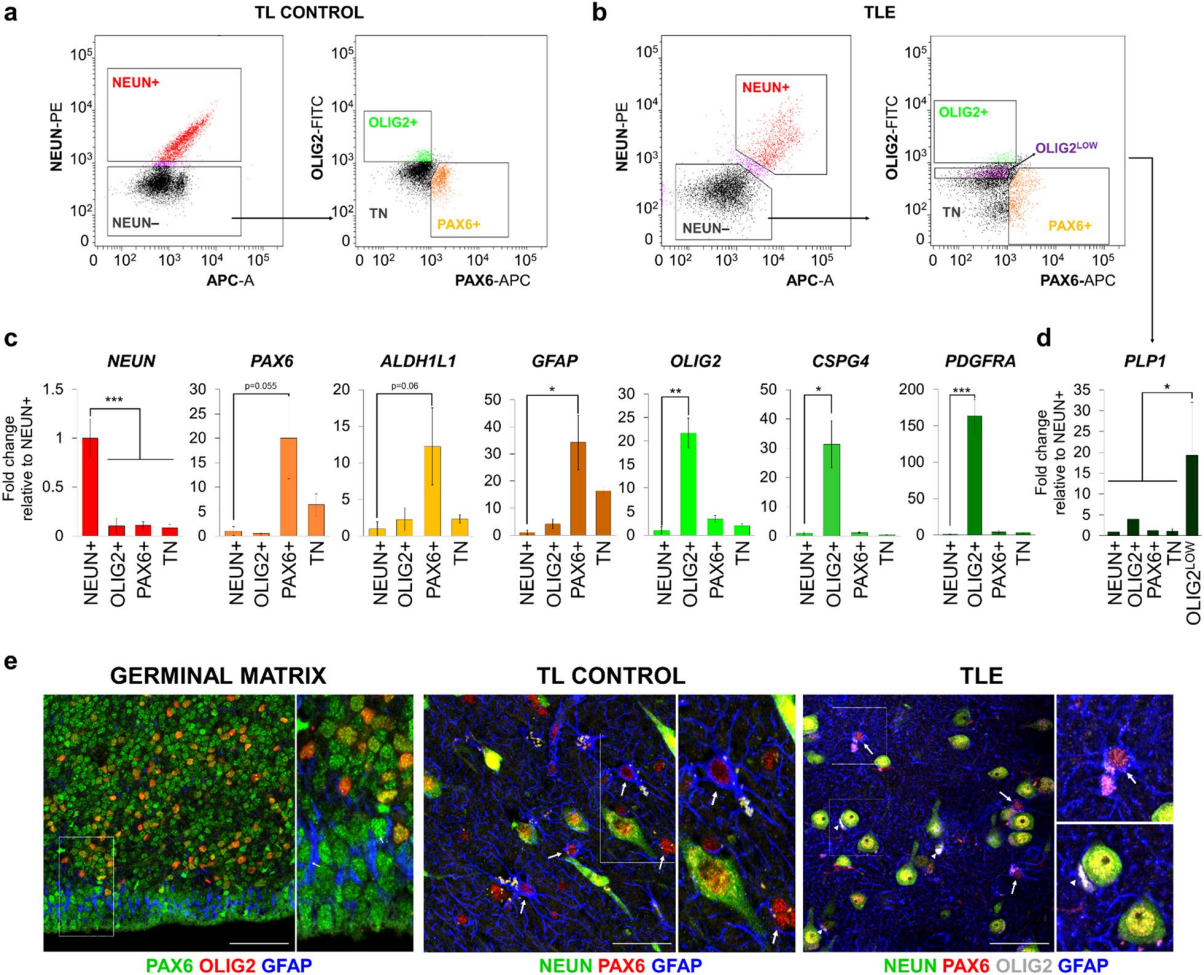
Simultaneous isolation of astrocyte, neuronal, and OPC-enriched nuclei from human temporal neocortex **(a-b)** Fluorescence-activated nuclei sorting (FANS) using anti-NEUN, anti-PAX6, and anti-OLIG2 antibodies simultaneously isolates three distinct nuclei populations from human postmortem control (TL) and epilepsy (TLE) temporal lobe neocortex: NEUN + , PAX6 + ( NEUN–), and OLIG2 + ( NEUN–), excluding the OLIG2^LOW^ fraction. TN = triple negative (NEUN–PAX6–OLIG2–) population. See also Additional file 1: Fig. S1a. **(c)** Gene expression analysis by RT-qPCR confirms high expression of the genes used as markers for isolation and shows significantly enriched expression of the astrocytic markers *GFAP* and *ALDH1L1* in PAX6 + ( NEUN–) nuclei and of the OPC markers *CSPG4* (NG2) and *PDGFRA* in OLIG2 + ( NEUN–) nuclei (n = 4 TL control brains). Bars represent mean ± SEM. P-values calculated from one-tailed t-test compared to NEUN + population. PAX6 + vs. NEUN + : *PAX6* p = 0.055; *ALDH1L1* p = 0.067; *GFAP* p = 0.019. OLIG2 + vs. NEUN + : *OLIG2* p = 0.0014; *CSPG4* p = 0.012; *PDGFRA* p = 0.0008. **(d)** Quantification of expression of the myelinating oligodendrocyte marker *PLP1* by RT-qPCR, showing its significantly higher expression in the OLIG2^LOW^ gated nuclei population compared to all others. Bars represent mean ± SEM, (n = 3 TLE brains). *p < 0.05 one-tailed t-test. **(e)** Representative immunofluorescence images of PAX6, OLIG2, and NEUN expression in developing germinal matrix (left), adult postmortem TL neocortex (center) and adult TLE neocortex (right). In adult TL and TLE neocortex, PAX6 expression is seen in GFAP + astrocytes (arrows) and NEUN + neurons but is absent in OLIG2 + oligodendroglial cells (arrowheads). Scale bar = 50 µM. See also Additional file 1: Fig. S1e

We then isolated nuclei from non-diseased (control) postmortem temporal neocortex (TL) containing gray and white matter, performed FANS using the three positive selection TF markers, and detected reliable separation of NEUN + , PAX6 + / SOX9 + (NEUN–OLIG2–), and OLIG2 + (NEUN–PAX6–/SOX9–) populations, hereafter referred to as NEUN + , PAX6 + / SOX9 + , and OLIG2 + for simplicity (Fig. 1a, Additional file 1: Fig. S1a, b). Analysis of canonical lineage-specific markers in each of these populations by RT-qPCR confirmed strong enrichment of neuronal, astrocytic, and OPC markers in the respective populations. PAX6 + nuclei were distinctly enriched for the astrocytic markers *ALDH1L1* and *GFAP*, as well as for *PAX6* (Fig. 1c, Additional file 1: Fig. S1c, d). SOX9 + nuclei were also enriched for astrocytic markers, but to a slightly lesser extent (Additional file 1: Fig. S1d), prompting subsequent FANS experiments to be performed with PAX6 only. OLIG2 + nuclei were enriched for the OPC markers *OLIG2*, *CSPG4* (NG2) and *PDGFRA* (Fig. 1c), and, importantly, showed low expression for the myelinating oligodendrocyte marker *PLP1* (Fig. 1d, Additional file 1: Fig. S1c). Instead, *PLP1* expression was enriched in a distinct population of nuclei derived from gating on low OLIG2 expression (OLIG2^LOW^) (Fig. 1b, d; Additional file 1: Fig. S1c). This suggested that excluding the OLIG2^LOW^ fraction of the OLIG2 + population (as shown in Fig. 1b and Additional file 1: Fig. S1a) can enrich for OPCs, relative to myelinating oligodendroglial populations, and this gating strategy was used for all subsequent sorting experiments. Finally, the NEUN–PAX6–OLIG2– triple negative (TN) population was depleted of astrocyte, OPC, and neuronal markers but showed strong enrichment for the microglial marker *CD11b* (Fig. 1c, Additional file 1: Fig. S1c).

We also characterized the cell type-specific distribution of OLIG2, PAX6, and NEUN protein expression in situ, in adult TL and TLE neocortex, as well as in developing brain as a control. PAX6 was expressed widely during neurodevelopment in human anterior germinal matrix tissue (18–20 gestational weeks), where it co-localized with presumed GFAP + radial-like glia and OLIG2 + glial progenitors (Fig. 1e). NEUN expression, as expected, was negative in the developmental germinal matrix (data not shown). In adult tissues, PAX6 was expressed strongly in (GFAP +) astrocytes in both normal adult and epileptic adult temporal lobe neocortex, and its expression did not appear to overlap with OLIG2-positive cells, as seen during development (Fig. 1e). In adult tissues, NEUN was expressed exclusively in neurons and OLIG2 was expressed by PAX6– glia in both TL control (Additional file 1: Fig. S1e) and TLE tissues (Fig. 1e). Interestingly, while NEUN + nuclei showed very low expression of *PAX6* (Fig. 1c), in line with previous human transcriptome studies [46], we detected weak PAX6 immunoreactivity in adult TL neurons (Fig. 1e). This discrepancy did not affect the FANS isolation strategy, since PAX6 + astrocytes were isolated from the NEUN– fraction. Overall, the gene and protein expression patterns of NEUN, PAX6, and OLIG2 provided confidence in their use for simultaneous isolation of adult NEUN + neuronal, PAX6 + (NEUN–) astrocyte, and OLIG2 + OPC-enriched nuclei populations.

### Nuclear RNA-seq validates FANS cell type specificity

To further validate this FANS isolation strategy, we analyzed the full nuclear transcriptome of NEUN + , PAX6 + , and OLIG2 + nuclei populations isolated from non-diseased fresh-frozen human postmortem TL and from pathological TLE neocortex, both containing gray and white matter (Additional file 2: Table S1). We focused specifically on the less well-characterized human neocortex (rather than hippocampus) of TLE samples, which contained diffuse subpial (Chaslin) and neocortical astrogliosis on diagnostic neuropathology, including away from sites of electrode placement, but lacked other lesional pathology (Table 1). All TLE neocortical samples used for sequencing had recent electrode-recorded primary seizure focus. Given the postmortem nature of TL specimens, we employed cDNA synthesis and library preparation kit optimized for partially degraded RNA with simultaneous depletion of ribosomal RNA, which passed quality control requirements.

**Table 1:** Sample information

Sample ID	Tissue, location	Age, Gender PMT	NUCLEI RNA-SEQ	SC RNA-SEQ	Pathological diagnosis
**Cell-type specific FANS Validation, TL vs. TLE**					
10662	TL control, temporal lobe neocortex	34yo, M 12 h PMT	✓		No neuropathological changes seen in cortex
10355	TL control, temporal lobe neocortex	45yo, F 12 h PMT	✓		No neuropathological changes seen in cortex
10997	TL control, temporal lobe neocortex	27yo, M 21 h PMT	✓		No neuropathological changes seen in cortex
12321	TLE, temporal lobe neocortex	50yo, M	✓		Cortical and Chaslin Gliosis; White matter neuronal heterotopia
10308	TLE, temporal lobe neocortex	29yo, F	✓		Cortical and Chaslin gliosis; FCD Ia
12726	TLE, temporal lobe neocortex	13yo, M	✓		Cortex without pathologic changes; *
14431	TLE, temporal lobe neocortex	28yo, M		✓ (10X, v2)	Cortical Gliosis; Ectopic white matter neurons with mild hypertrophy;
19619	TLE, temporal lobe neocortex	31yo, F		✓ (10X, v2)	Chaslin gliosis; *
20188	TLE, temporal lobe neocortex	58yo, F		✓ (10X, v3)	Cortical and Chaslin gliosis, rare neurons with hypertrophy and disoriented dendrites; *
12814	TLE, temporal lobe neocortex	11yo, M		(10X, v1)	Chaslin gliosis; *
13059	TLE, temporal lobe neocortex	8yo, M		✓ (10X, v1)	Gliosis, neuronal heterotopia;
**Phenotypic analysis of epilepsy glia**					
12321	TLE, temporal lobe neocortex	50yo, M	✓’ (high)	✓	Cortical and Chaslin Gliosis; White matter neuronal heterotopia;
10308	TLE, temporal lobe neocortex	29yo, F	✓ (low)	✓	Cortical and Chaslin gliosis; FCD Ia;
12726	TLE, temporal lobe neocortex	13yo, M	✓’ (high)		Cortex without pathologic changes; *
14431	TLE, temporal lobe neocortex	28yo, M	✓(low)	✓	Cortical Gliosis; Ectopic white matter neurons with mild hypertrophy;
12319	Epilepsy, frontal lobe neocortex	27yo, M	✓’ (high)		Chaslin Gliosis; Ischemic changes
12433	Epilepsy, TS, frontal lobe neocortex	4yo, M	✓ (low)	✓	Cortical and Chaslin Gliosis; **

*PMT* Postmortem time, *IF* Immunofluorescence, *M* Male, *F* Female, *yo* years old; *h* hours; *TS* Tuberous sclerosis. *FCD* Focal cortical dysplasia. * Hippocampal sclerosis seen away from the sampled area; ** tubers seen away from the sampled area.’ Samples included in the IF analysis of Ki67 proliferation cell type distribution

**Fig. 2:**
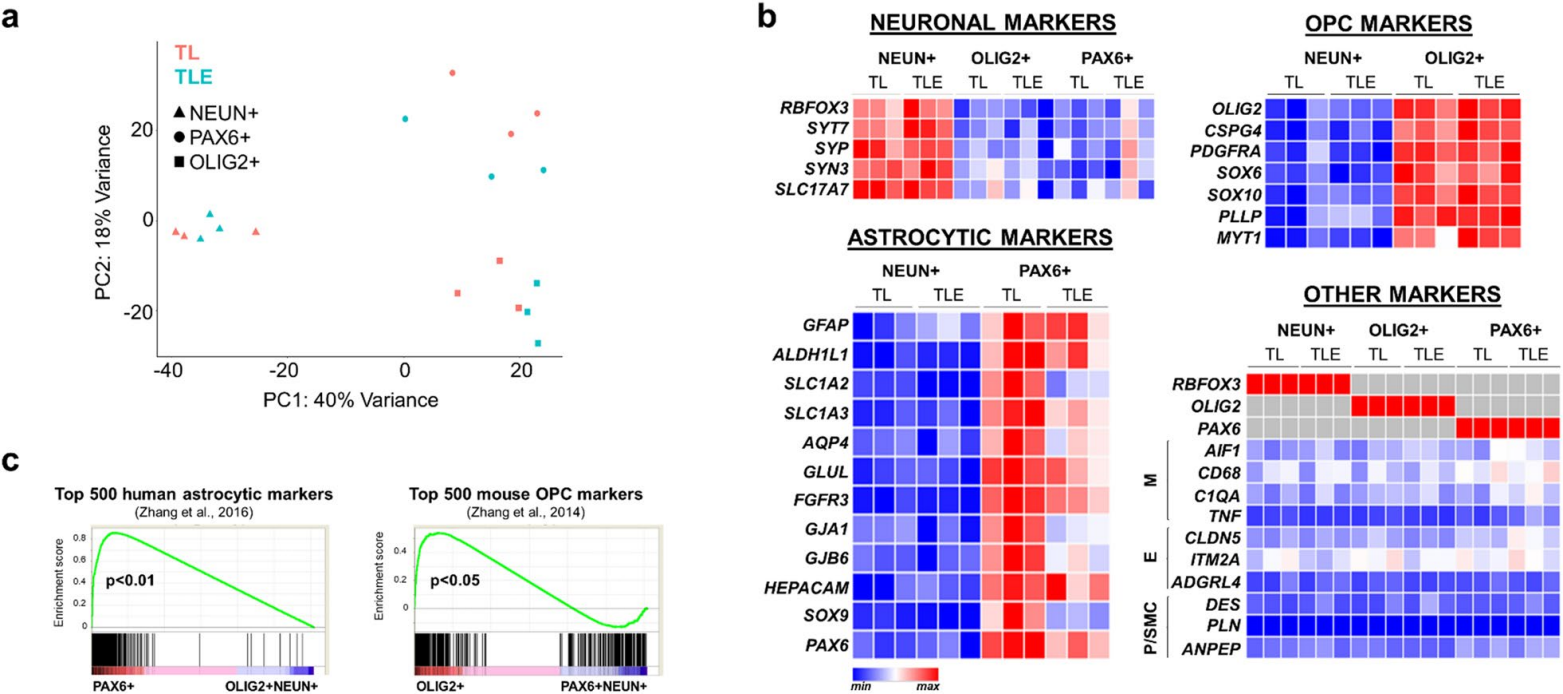
Nuclear RNA-seq confirms cell type specificity in TL and TLE FANS-isolated populations. **(a)** RNA-seq principal component analysis reveals separation driven by sorted cell type. **(b)** Heatmap representations of nuclear gene expression (rld-normalized) RNA-seq data, derived from sorted PAX6 + , NEUN + , and OLIG2 + populations. Strong and selective enrichment of astrocytic, neuronal, and OPC markers is seen in each respective FANS population (normalized by row), with lack of contaminant microglial (M), endothelial (E), and pericyte / smooth muscle cell (P/SMC) gene expression in the sorted populations (normalized by column). **(c)** Gene set enrichment analyses (GSEA) confirm significant, cell type-specific enrichment. Rld-normalized nuclei RNA-seq data (PAX6 + , OLIG2 + , NEUN +) is analyzed against the top 500 overexpressed set of genes unique to resting human astrocytes [46] or to mouse OPCs [52]. For each gene set, each gene expression value is calculated relative to the average expression of all other populations and then sorted by highest relative expression

We first performed unsupervised clustering and principal component analysis (PCA) of all sequenced samples, which separated according to cell type (Fig. 2a). As expected, glial populations (astrocytes and OPCs) were more similar to one another than to neuronal populations in the first PC dimension, and astrocytes separated from OPCs in the second PC dimension, in both TL and TLE tissues (Fig. 2a). Analysis of canonical lineage-specific markers corroborated the specificity of our isolation technique for astrocyte and OPC populations in both TL and TLE tissue types, despite notable downregulation of several canonical astrocyte markers in the epilepsy samples (Fig. 2b). PAX6 + nuclei populations were strongly enriched for astrocyte markers, while OLIG2 + nuclei were enriched for OPC and pan-oligodendroglial markers; and both showed minimal expression of vascular, inflammatory, and neuronal-specific markers (Fig. 2b). Using gene set enrichment analysis (GSEA) [33], we also compared how TL control FANS nuclear transcriptomes relate to previous whole cell transcriptome data obtained from purified human astrocytes [46] and mouse OPCs [52]. We found significant enrichment of the top HepaCAM-purified human astrocyte genes [46] within PAX6 + transcriptomes (Fig. 2c), and of the top mouse OPC genes [52] within OLIG2 + transcriptomes (Fig. 2c). Thus, nuclear RNA-seq defined distinct cell type-specific transcriptome signatures in the three sorted populations and corroborated the concordance between nuclear and whole cell RNA for highly expressed and cell lineage-specific transcripts, as previously demonstrated in other systems [53–55]. Importantly, the analysis provided confidence in using this immunotagging strategy for further analysis of cell type-specific transcriptome dysregulation in the context of epilepsy.

### Functional enrichment analysis of dysregulated genes in human TLE cell types

**Fig. 3:**
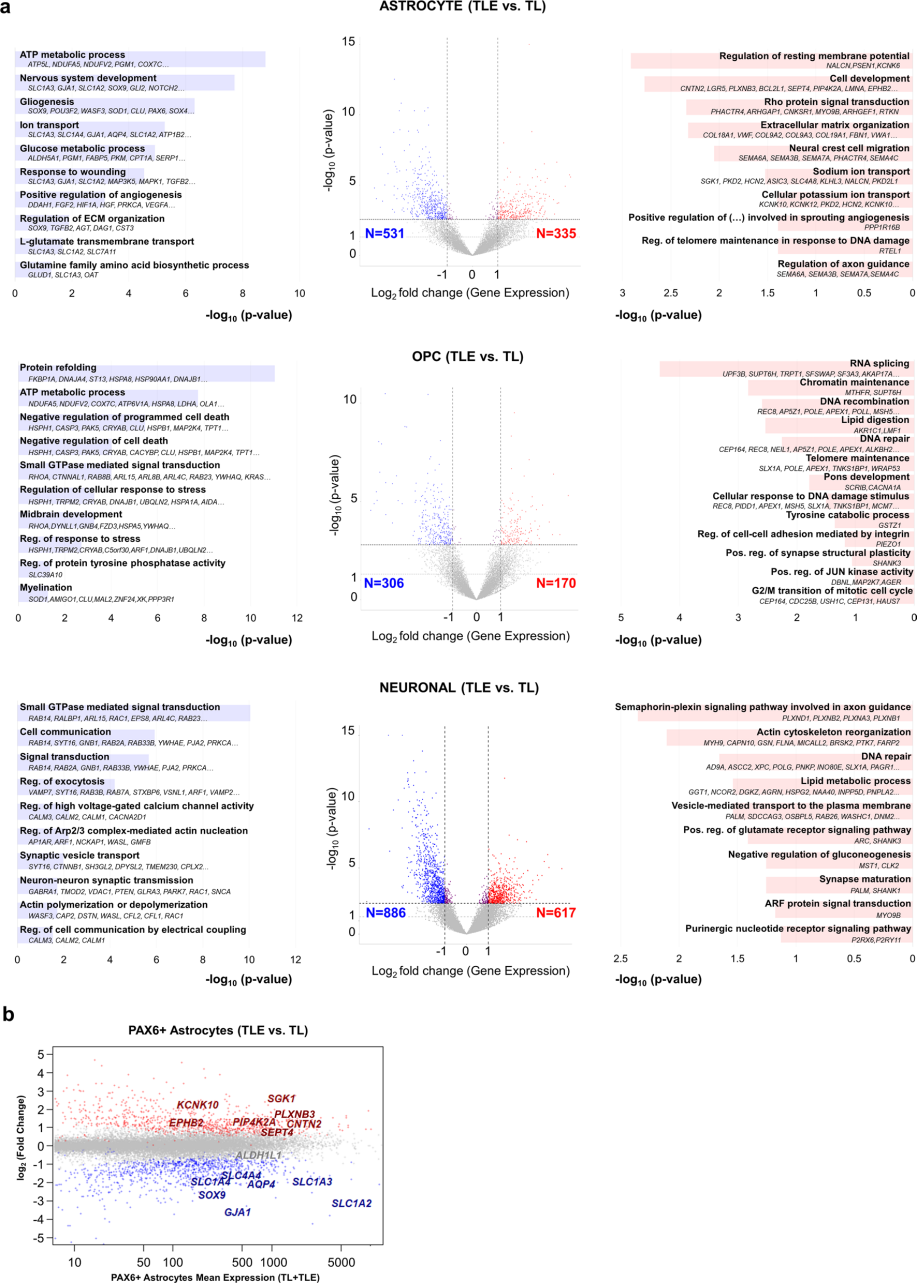
Dysregulated genes and biological processes in human temporal lobe epilepsy astrocyte, OPC and neuronal populations. **(a)** Differential expression analysis (TLE vs. TL) for each sorted cell type is represented by a volcano plot, depicting significantly upregulated (red) and downregulated (blue) genes in the epilepsy astrocyte, OPC, and neuronal populations. Functional enrichment analyses, performed using HOMER and DAVID (D) tools, depict top-enriched biological processes dysregulated in epilepsy, for each cell type (log_2_ fold change < -1 for downregulated and > 1 for upregulated, Benjamini–Hochberg adjusted p-value < 0.1). See Additional file 4: Table S3, Additional file 5: Table S4 for complete list. **(b)** MA plot shows the relative expression of genes differentially up- and down-regulated in PAX6 + epilepsy astrocytes, marked by red and blue dots, respectively

Next, we performed a series of differential transcriptome analyses (TLE vs. TL) in order to define the epilepsy-specific and cell type-specific dysregulated genes in TLE astrocytes, OPCs, and neurons (Additional file 3: Table S2), and used functional enrichment to characterize the most significantly enriched biological processes defined by up- and downregulated genes in each TLE cell type (Fig. 3a, Additional file 4: Tables S3, Additional file 5: Table S4). In general, we observed a net loss of gene expression in epilepsy compared to postmortem control, in each cell type population, detecting a larger number of downregulated than upregulated genes. In epilepsy astrocytes (Astrocyte TLE vs. Astrocyte TL control), differentially downregulated genes related to mature astrocyte function (such as “L-glutamate transmembrane transport”, “glucose metabolic process”, and “response to wounding”) while upregulated genes related to development and potassium ion transport, among others (Fig. 3a, Additional file 4: Table S3, Additional file 5: Table S4). In epilepsy OPCs, differentially upregulated genes were similarly enriched for terms related to progenitor development and proliferation while myelination and stress-related genes were downregulated (Fig. 3a, Additional file 4: Table S3, Additional file 5: Table S4). Differentially dysregulated genes in neurons (TLE vs. TL) were highly significant for GO terms related to cell communication, signal transduction, and axon guidance (Fig. 3a, Additional file 4: Table S3, Additional file 5: Table S4). The majority of differentially expressed genes in these analyses were cell-unique as they were not significantly dysregulated in the other cell type comparisons (Additional file 4: Table S3). Excluding the small subset of non-unique differential genes did not significantly alter the top enriched functional biological processes in each dataset analysis (Additional file 4: Table S3).

Overall, these enrichment analyses recapitulated cell type-specific processes related to astrocyte, OPC, and neuronal function previously established in mouse models, and also uncovered several still poorly understood glial-specific pathological changes in the context of human TLE. One striking example was the significant alteration in the phenotype of epilepsy astrocytes towards de-differentiation, with downregulation of genes important for normal maintenance of synaptic homeostasis, and glial differentiation. Among the most robustly, significantly and uniquely downregulated genes in epilepsy astrocytes were the glutamate transporter GLAST/EAAT1 (*SLC1A3*); the sodium-dependent neutral amino acid transporter ASCT1 (*SLC1A4*); the gap junction proteins connexin 30 (*GJB6*) and connexin 43 (*GJA1*); and the water channel *AQP4* (Fig. 3b). GLT-1/EAAT2 (*SLC1A2*) was significantly downregulated in both TLE astrocytes and TLE neurons, with the relative expression of *SLC1A2* in astrocytes being much higher than in neurons (Additional file 2: Table S1, Additional file 3: Table S2). Important genes during astrocytic differentiation, such as *SOX9*, were also observed downregulated in TLE tissues. In contrast, significantly and uniquely upregulated genes in TLE astrocytes included many related to ECM organization and cell development (Fig. 3b, Additional file 2: Table 1, Additional file 3: Table S2, Additional file 4: Table S3 , Additional file 5: Table S4), overall pointing towards a phenotypic switch in epilepsy astrocytes from mature synaptic maintenance to extracellular remodeling, with possible tendency towards immature de-differentiation.

### Single cell RNA-seq uncovers TLE glial subpopulations with aberrant OPC-like signatures

**Fig. 4:**
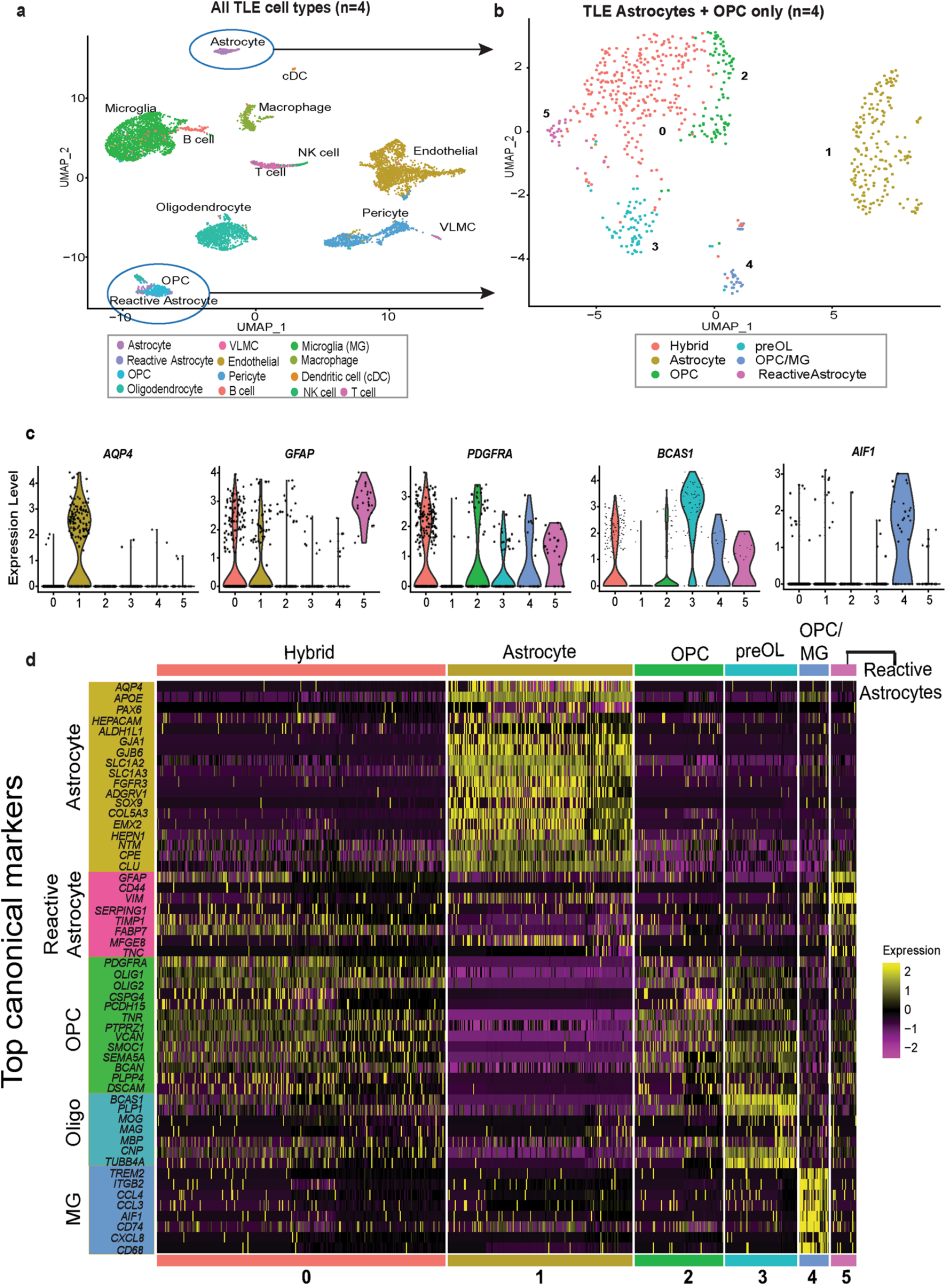
Single-cell transcriptomics of human temporal lobe epilepsy reveals mixed lineage glial subpopulations. **(a)** Uniform Manifold Approximation and Projection (UMAP) plot for integrated TLE samples from four different patients (14431, 13059, 19619, and 20188; see Table1). Clusters are colored by annotated cell types and indicated by labels. See also Additional file 7: Fig. S2a. **(b)** UMAP plot of subclustered astrocyte and OPC subpopulations from (a) after reclustering. Glial subpopulations cluster in six distinct clusters (0–5), colored by cluster ID. **(c)** Violin plots of log-normalized gene expression for canonical cell type-specific glial markers across the six subclusters identified in (b): *AQP4* (Astrocyte), *GFAP* (Reactive astrocyte), *PDGFRA* (OPC), *BCAS1* (premyelinating oligodendrocyte) and *AIF1* (Microglia). Hybrid cluster 0 shows elevated expression of both *PDGFRA* and *GFAP*. **(d)** Heatmap of log-normalized and z-scored gene expression data from the subclustered TLE glia object, plotting canonical cell-type lineage markers for Astrocytes, Reactive astrocytes, OPC, Oligodendrocytes (Oligo), and microglia (MG) across glial subclusters 0–5, highlighting dual OPC/reactive astrocyte signatures in hybrid cluster 0. See also Additional file 7: Fig. S2b (same analysis plotting top 10 differentially expressed genes per cluster)

To further scrutinize the heterogeneity of glial cells in epilepsy, we performed single cell RNA-seq (scRNA-seq) on fresh epilepsy temporal neocortex derived from five patients with medically refractory TLE, again prioritizing tissue from electrode-recorded primary seizure focus. Overall, we sequenced a total number of 17,057 single cells, with an average depth of 126,498 reads/cell (Additional file 6: Table S5). The raw data was processed with the Cell Ranger pipeline (10X Genomics, v6.1.1) [34]. Samples with acceptable quality control metrics (14431, 13059, 20188 and 19619, see Methods and Additional file 6: Table S5) were used for downstream computational analyses using Seurat [35, 56]. Integration of scRNA-seq TLE data showed clustering of all four samples by distinct cell types (Fig. 4a) rather than by age or patient origin, with contribution of each patient sample to all cell type clusters (Additional file 7: Fig. S2a). Using canonical cell-type specific markers [57–60] and validated gene expression data from adult human brain (Allen brain atlas [38]), we annotated distinct subpopulations of astrocytes, OPCs, myelinating oligodendrocytes, microglia, macrophages, T- and B-cell lymphocytes, NK cells, dendritic cells (DC), endothelial cells, pericytes, and vascular leptomeningeal cells (VLMC) within the integrated TLE scRNA-seq dataset (Fig. 4a, Additional file 8: Table S6). In contrast to our FANS RNA-seq dataset generated from frozen tissue, neurons were underrepresented in the TLE scRNA-seq dataset, likely due to increased sensitivity to hypoxia during fresh tissue processing.

We focused subsequent analyses on OPCs and astrocytes, the two main glial subpopulations of interest in this study, which were well resolved in the scRNA-seq dataset. Astrocytes showed strong differential expression of canonical astrocyte markers such as *AQP4*, *SLC1A2*, *GJB6*, and *SOX9*; while OPCs were defined by top differential markers such as *PDGFRA*, *CSPG4*, and *OLIG2* (Fig. 4, Additional file 8: Table S6). Interestingly, markers for reactive astrocytes (*GFAP, VIM, CD44*) [57–59] identified a small population of cells next to the OPC cluster, and away from the main astrocyte cluster, indicating molecular heterogeneity between the two types of astrocyte populations (Fig. 4a). To further elucidate the relationship between the different astrocyte subpopulations and OPCs, we isolated these populations for further analysis at a higher resolution (Fig. 4b). Iterative clustering at various resolutions confidently resolved six clusters with distinct gene expression profiles, including *AQP4* + and PAX6 + astrocytes (cluster 1), *PDGFRA* + and *OLIG2* + OPCs (cluster 2), *BCAS1* + oligodendroglia (preOL, cluster 3), *GFAP* + *VIM* + reactive astrocytes (cluster 5), as well as two populations with non-canonical signatures (cluster 0 and 4) (Fig. 4b-d, Additional file 7: Fig. S2b and Additional file 9: Fig. S3a-e). The first and most prominent non-canonical population (cluster 0, referred to as Hybrid from here on), was situated between OPC and reactive astrocytes (Fig. 4b), and displayed hybrid expression of both canonical OPC marker genes (such as *PDGFRA* and *OLIG2*) and reactive astrocyte marker genes (such as *GFAP, FABP7*, and *TIMP1*) (Fig. 4c-d, Additional file 7: Fig. S2b-c and Additional file 9: Fig. S3b, c). The second and minor non-canonical population displayed dual expression of OPC markers (including *PDGFRA* and *OLIG2*) and microglial markers (such as *AIF1*, *CD68*, *CCL4*) (cluster 4, referred to as OPC/MG from here on) (Fig. 4c, d, Additional file 7: Fig. S2b, c, Additional file 9: Fig. S3c, d). Of note, OPC/MG cells were part of the OPC population in the main integrated object, where they clustered distinctly from other inflammatory cells and expressed lower levels of canonical monocyte markers (such as *AIF* and *CX3CR1*) compared to the main microglia and macrophage clusters (Additional file 9: Fig. S3f). Rare populations of premyelinating oligodendroglia (preOL) marked by strong BCAS1 were also detected in this subclustered analysis (Fig. 4c-d, Additional file 9: Fig. S3e), a population recently described in the gray matter of normal adult cortex and in multiple sclerosis lesions [61] which tends to cluster more closely with OPCs than with mature myelinating oligodendrocytes (Ramos et al., in revisions).

**Fig. 5:**
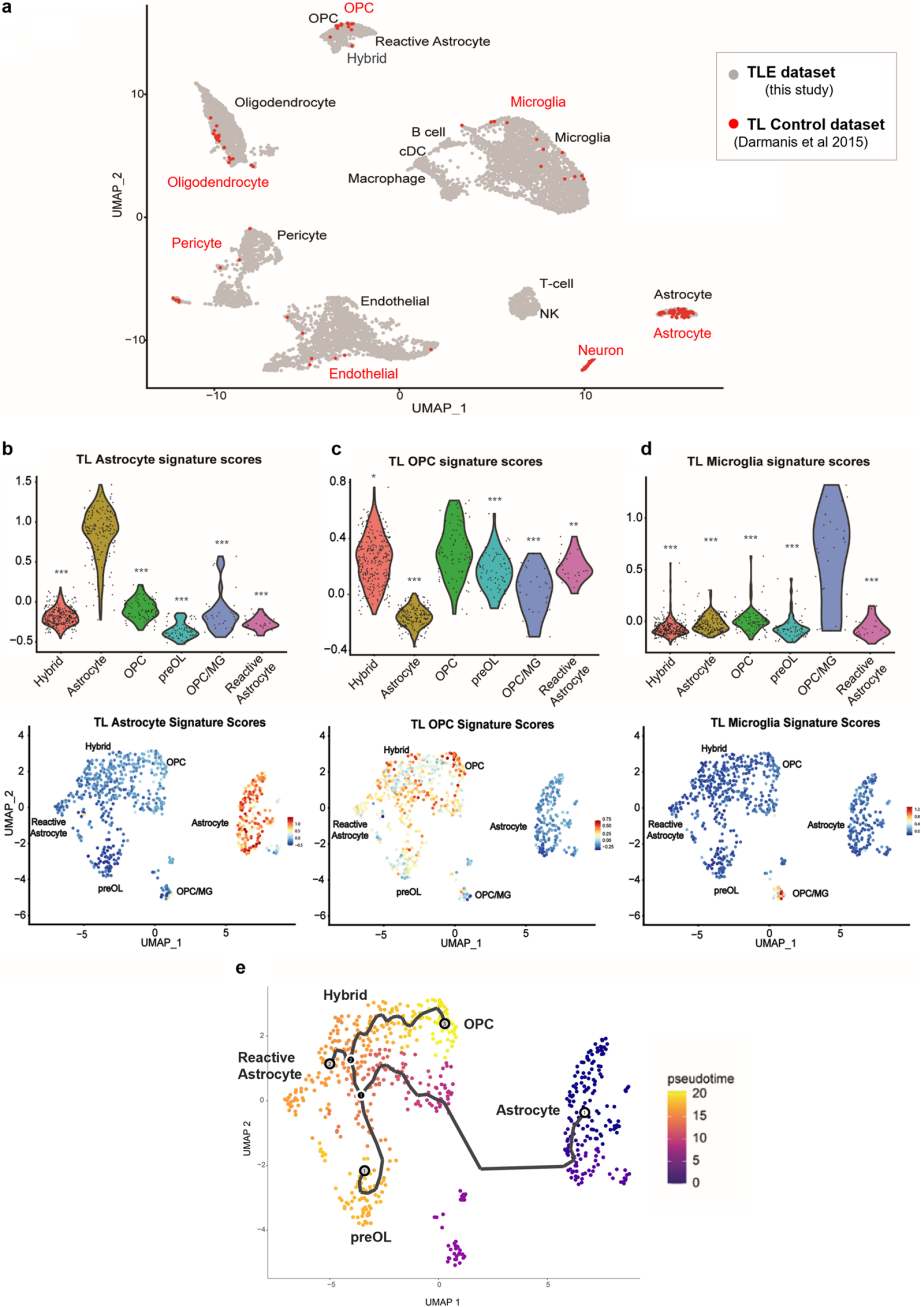
Comparison of normal and epilepsy temporal lobe scRNA-seq datasets confirms aberrant glial phenotypes. **(a)** UMAP representation of TLE data from Fig. 4a integrated with normal TL single-cell RNAseq data from Darmanis et al., *PNAS* 2015 [39]. Normal TL is denoted in red and TLE in gray. **(b-d)** Violin plots (top) and scaled gradient feature plots (bottom) representing projections of normal “TL astrocyte” (b), normal “TL OPC” (c) and normal “TL microglia'' (d) signature scores onto the diseased TLE astrocyte and OPC subclustered dataset in Fig. 4b, depicting abnormal enrichment of OPC-like signatures in TLE hybrid glia and reactive astrocytes (* = p-adj. < 0.05; ** = p-adj. < 0.005; *** = p-adj. < 0.0005 using Wilcoxon rank test, with Benjamini Hochberg correction for multiple hypothesis testing). **(e)** UMAP representation of Monocle3 pseudotime lineage trajectory analysis of TLE subclustered glia shown with astrocyte as the root cluster, depicting greater pseudotime similarity between OPCs, hybrid glia, and reactive astrocytes compared to astrocytes. See also Additional file 10: Fig. S4

Next, we asked if the discovered hybrid populations are exclusively present in epileptic TLE samples. We used a previously published normal temporal lobe (TL) scRNA-seq dataset [39], similarly derived from surgical samples and processed for whole single cell RNA-seq, and compared it to our TLE scRNA-seq data. TL and TLE datasets were integrated using Seurat [35, 56]. Integrated dimensionality reduction and cell-type annotation analysis revealed all TLE cell types clustering with their respective normal TL counterparts, except for TLE reactive astrocytes and TLE hybrid glia, both of which clustered with normal TL OPCs (Fig. 5a, Additional file 10: Fig. S4a, b). To quantitate these observations, we projected normal TL astrocyte and OPC gene expression signatures onto the epilepsy data and calculated normal “TL Astrocyte'' and normal “TL OPC” signature enrichment scores for all TLE subpopulations (Fig. 5b-d). Normal “TL Astrocyte” signature was highly enriched in the TLE astrocyte population only, with high significance compared to all other populations (Fig. 5b). In contrast, normal TL OPC signature was not only enriched in TLE OPCs but also in the Hybrid, reactive astrocyte, preOL, and OPC/MG TLE populations, albeit to a lesser extent as compared to TLE OPCs (Fig. 5c). We also calculated normal “TL Microglia” and normal “TL oligodendrocyte” scores and projected them onto TLE glia (Fig. 5d, Additional file 10: Fig. S4c). This revealed highest “TL Microglia'' enrichment score in the TLE OPC/MG cluster (Fig. 5d) and highest “TL Oligodendrocyte” enrichment score in the TLE preOL cluster (Additional file 10: Fig. S4c), in agreement with our cell type annotations. Overall, projecting normal adult temporal lobe cortex cell type-specific gene expression signatures onto our epilepsy dataset confirmed the presence of TLE glial subpopulations with an aberrant, mixed-lineage phenotype, including a major population with an OPC/Reactive astrocyte hybrid signature, and a minor population with OPC/microglia hybrid signature.

Recent advances in trajectory analysis enable inferences of lineages on a pseudotime trajectory to better understand cell transition states [40, 62, 63]. To explore the relationship between the delineated TLE glial subpopulations in the context of their potential lineage states, we constructed trajectories in our TLE subclustered object using Monocle3. The Monocle3 algorithm learns a sequence of gene expression changes in cells and places each cell on a trajectory as a function of pseudotime [40]. We performed the analysis multiple times, choosing the root node as either astrocyte, reactive astrocyte, or OPC, to understand where hybrid cells are in relation to the root node in pseudotime. OPCs were always furthest away from canonical astrocytes in pseudotime, with hybrid and reactive astrocytes showing an intermediate pseudotime much closer to OPCs than to astrocytes, regardless of the root node chosen (Fig. 5e, Additional file 10: Fig. S4d). Overall, both TLE hybrid cells and reactive astrocytes exhibited more pseudotime similarity to OPCs than to resting astrocytes, suggesting their lineages may be more closely related.

### A subset of GFAP + TLE glia displays an aberrant proliferative phenotype

**Fig. 6:**
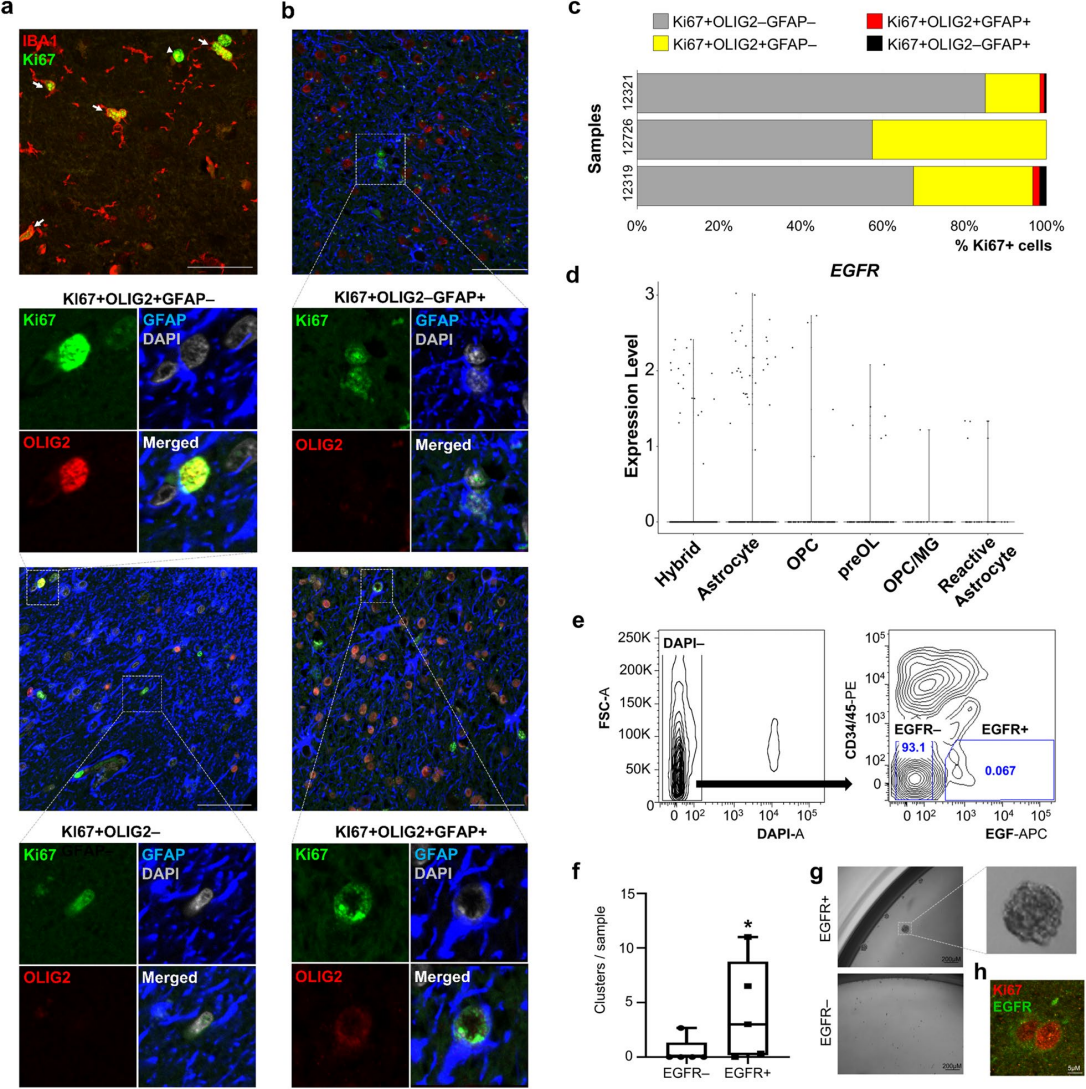
Glial proliferation in human temporal lobe epilepsy. **(a-b)** Representative immunofluorescence images from the subset of TLE samples with high proliferation, as assessed by Ki67 labeling. A large portion of Ki67-positive cells co-express the microglial marker IBA1 (arrows), although Ki67 + IBA1**–** cells are not uncommonly seen as well (arrowheads) (a). Representative immunofluorescence images of GFAP, OLIG2 and Ki67 co-expression in TLE tissue (b). **(c)** Quantification of cell type-specific contribution to proliferation in the subset of TLE samples with high proliferation index, assessed by co-expression of Ki67 with OLIG2 and/or GFAP. Ki67 + cells were examined within the entire TLE tissue section, 14–58 40X fields overall. **(d)** Violin plot of log-normalized *EGFR* expression across the six TLE glial subpopulations from Fig. 4b. **(e)** FACS plots showing isolation strategy to purify EGFR + (CD34–CD45–) cells from fresh TLE tissue. A small but distinct population of EGFR + (DAPI–CD34–CD45–) cells are isolated, and subjected to primary cell culture along with EGFR– cells. FACS excludes dead cells (DAPI +), endothelial (CD34 +) and inflammatory (CD45 +) cells from the culture analysis. Numbers in boxes indicate % from total DAPI– cells. **(f)** EGFR + cells form proliferative clusters in vitro, significantly more compared to their EGFR– counterpart, when grown under low-adherence neural stem cell condition medium (n = 5 TLE samples, 10cells/µl (2000 cells/well); 1–3 wells per sample, week 2). Box-plots represent median, minimum and maximum value. P-value calculated using one-tailed U-Mann Whitney non-parametric test. **(g)** Representative microscope images (10X) of proliferative clusters growing from TLE EGFR + cells. **(h)** Human TLE tissue immunofluorescence demonstrates occasional co-localization of the rare EGFR + cells with the proliferative marker Ki67. In (a–b) nuclei are counterstained with DAPI. Scale bar = 50 µM unless otherwise specified

To confirm the presence of GFAP + OLIG2 + glia in human TLE samples at a protein level, we performed immunofluorescence analysis in primary TLE tissue using anti-OLIG2, and anti-GFAP antibodies. We also included anti-Ki67 antibody in the experiment to evaluate the proliferative status of this hybrid population, given its immature transcriptome signature. In half of TLE samples, we detected higher degree of cell proliferation exhibited by Ki67 immunoreactivity (Fig. 6a-c, Table 1), than expected for the largely quiescent normal TL parenchyma [64]. Within the pool of proliferative Ki67 + TLE cells, a small subset was indeed double positive for GFAP and OLIG2 (Fig. 6b, c), corroborating the presence of GFAP + OLIG2 + hybrid glia in human TLE tissue, with proliferative potential. Intriguingly, a few Ki67 + cells were GFAP + astrocytes (Ki67 + GFAP + OLIG2-) (Fig. 6b, c), which are typically quiescent under physiological conditions [64, 65]. As expected, the majority of the remaining Ki67 + cells corresponded to microglia (IBA1 +), and proliferative OPCs (Ki67 + OLIG2 + GFAP-) (Fig. 6a-c). IBA1 + OLIG2 + cells were not detected.

Finally, to study functionally the proliferative properties of TLE glia, we employed an EGF-based purification strategy which has been used previously to isolate stem cell astrocytes from the adult rodent subventricular zone [66] and proliferative human stem cell populations from germinal matrix and glioblastoma fresh tissue samples [41, 42, 67]. A quick analysis of the scRNA-seq data showed that EGFR expression was high primarily in a subset of cells from the astrocyte and hybrid subclusters (Fig. 6d). Using EGF as a positive EGFR selection marker and CD34/45 to exclude endothelial and inflammatory cells, we detected a small but distinct population of EGFR + (CD34–CD45–) cells (Fig. 6e) from five different pathological epilepsy tissues (Table 1), which we purified for downstream functional characterization of proliferation. Overall, EGFR + cells formed significantly more proliferative clusters than EGFR– cells under serum-free, stem cell medium conditions (Fig. 6f, g), corroborating in vitro the proliferative phenotype previously observed in vivo (Fig. 6b, c, h).

## Discussion

Recent advances in multi-omics technology have enabled deeper understanding of glial pathology in several brain disorders [68, 69]. While glia are the most abundant cell type in the adult brain, their contribution to epileptogenesis remains relatively less well characterized compared to that of neurons, in human diseased tissue [70]. In this study, we developed a new isolation strategy of tissue-derived astrocytes and OPCs and combined it with bulk and single cell transcriptomics to study glial biology in primary temporal lobe epilepsy surgical tissue. Our analyses capture a rich repertoire of unique nuclear transcripts within each population at high throughput and elucidate further the distinct transcriptome programs dysregulated in neurons, astrocytes, and OPCs in the context of medically refractory human TLE. We discover a shift from mature to progenitor-like phenotype in the transcriptome of TLE astrocytes, which we resolve further using independent single cell RNA-seq TLE dataset and validate in situ and in vitro using primary human samples. The findings motivate further studies into the dysfunctional role of reactive glia in temporal lobe epilepsy.

### Simultaneous isolation of human astrocyte, neuronal, and OPC-enriched populations by FANS

Recently, several methods have emerged for isolating resting astrocytes and oligodendroglia acutely from human or mouse brain by means of immunopanning [27, 46, 52]. Others have used the surface marker GLAST to isolate astrocytes by FACS [71], with some limitation on yield due to the sensitivity of GLAST + cytoplasmic processes to mechanical dissociation and enzymatic digestion. Nuclei isolation from snap-frozen tissue circumvents the problem of cytoplasmic cell–cell processes dissociation and minimizes transcriptome alterations or artifacts that may be incurred during purification or cell culture [72]. Previous attempts to characterize glia using this method have been largely limited to the analysis of NEUN– (“non-neuronal”) populations, which are fundamentally heterogeneous and include endothelium, pericytes, smooth muscle cells, microglia and other inflammatory cells, in addition to astrocyte and oligodendroglial lineages [25, 73–75]. Few studies have used OLIG2 or SOX10 to isolate oligodendroglial populations from the NEUN– fraction [76] (unpublished), and a single study has demonstrated the use of SOX9 to sort astrocyte nuclei from mouse brain [27]. No studies thus far have demonstrated the simultaneous isolation of two defined glial lineages, astrocytes and OPCs, from the NEUN– fraction. Given the pivotal role of astrocytes and OPCs in development and the increasing appreciation for their contribution to neurological dysfunction, the simultaneous FANS isolation of these two distinct glial subtypes from banked fresh-frozen brain tissue is a valuable resource for further glial-specific omics analyses in the context of various pathological human CNS disorders, especially when single nucleus transcriptomics is cost-prohibitive.

### An abnormal population of epilepsy glia show a hybrid state between OPCs and reactive astrocytes

Previous RNA-seq studies have begun to define the transcriptome in epilepsy mouse models and TLE patients [70, 77, 78], but glial contributions have not been thoroughly characterized. Our study used two independent analyses to characterize the transcriptome of human astrocytes and OPCs derived from medically refractory TLE samples: FANS nuclei RNA-seq using frozen tissue and single cell RNA-seq using fresh tissue. FANS RNA-seq analysis in epilepsy (vs. control) revealed a de-differentiation phenotype in both TLE OPCs and TLE astrocytes. TLE OPCs showed dysregulated functional pathways related to development, mitotic activity, cell–cell adhesion, TCA metabolism, and myelin sheathing, in line with their tendency to increase in number at the site of brain injury, whether through local proliferation or via migration [7–13]. TLE astrocytes showed significant downregulation of several mature function-defining genes, including the transporters *SLC1A3*, *SLC1A2,* and *SLC1A4* and the gap junction proteins connexin 30 (*GJB6*) and 43 (*GJA1)*, and upregulation of genes related to development and ECM / tissue repair. The observed dysregulation of glutamate, potassium ion channels, and connexin expression in human TLE astrocytes is consistent with prior rodent studies implicating astrocytic dysfunction in excitotoxicity and epilepsy [6, 79–86]. Using primary human samples, we further corroborated the observed progenitor-like transcriptomic phenotype in TLE glia by analyzing their proliferation activity, and found that a subset of them not only express Ki67 in-situ but also form abnormal proliferative clusters in-vitro.

Single cell transcriptomics confirmed enrichment of *PAX6* in astrocytes and *OLIG2* in OPCs, immunomarkers used in FANS, and further enabled analysis of glial heterogeneity at higher resolution than previously studied. By comparing epilepsy to normal glia signatures, using two different “control” single cell RNA-seq temporal lobe references [38, 39], we uncovered two abnormal populations of epilepsy glia, in addition to reactive astrocytes. The first is a rare population of OPCs with enriched inflammatory / microglial signature. Such populations have been recently described in multiple sclerosis [87, 88] and our study documents their presence in human epilepsy tissue as well. The second is a prominent population of TLE hybrid glia expressing both OPC and reactive astrocyte -associated genes, which represented the main focus of our downstream studies. Interestingly, TLE reactive astrocytes themselves were more OPC-like compared to astrocytes defined by canonical markers and they showed downregulation of canonical astrocyte markers as seen in the FANS analysis. Notably, many glia within the “hybrid” cluster and some within the “reactive astrocyte” cluster showed an aberrant GFAP + / OLIG2 + phenotype, the presence of which we corroborated further at the protein level in primary TLE samples. Glia with aberrant GFAP + / OLIG2 + phenotype were only resolved in our single cell analysis, likely due to the lower resolution of bulk RNA-seq FANS analysis but also possibility related to TLE sample heterogeneity. As these cells did not express high levels of *PAX6*, they were likely captured in our OLIG2 + FANS population. Combination of PAX6 + and OLIG2 + FANS for astrocyte and OPC enrichment followed by single cell sequencing may allow even higher resolution of glial subtypes in future studies.

In rodents, few prior studies have noted the occasional expression of Olig2 by astrocytes under physiological conditions [89–93]. Importantly, Olig2 is upregulated in (Gfap +) reactive astrocytes after brain injury, which renders cells more proliferative [94, 95]. Furthermore, Olig2 is an important driver of early glial development and astrocyte differentiation [96, 97], and in-vitro studies have shown the ability of astrocytes to transdifferentiate into OPCs [98] as well as the ability of OPCs to differentiate into type II astrocytes [99, 100]. While TLE hybrid glia similarly showed *GFAP* and *OLIG2* co-expression, and resembled reactive astrocytes in their signature profile and lineage analysis, they differed from previously reported Olig2-lineage astrocytes by their frequent expression of bona fide OPC gene markers, such as *PDGFRA* and *CSPG4*. Considering the plasticity and proliferative capacity of OPCs in the adult brain, we cannot rule out the possibility that at least some of the OLIG2 + GFAP + hybrid glia represent reactive OPCs. In all, our study does not definitively establish whether OLIG2 + GFAP + hybrid glia are astrocytes or OPCs, and Monocle trajectory analysis infers a continuous transition state in which OPCs, hybrid glia, and reactive astrocytes may be part of the same lineage. In the context of the above literature and our findings, we speculate two possible differentiation dynamics that involve a hybrid transition: 1) a subset of reactive TLE astrocytes de-differentiating into an OPC-like state or 2) a subset of reactive TLE OPCs transitioning into a reactive astrocyte-like state; the mechanics of these remain to be determined in future studies.

## Supplementary Information

**Additional file 1: Fig. S1**. Immunotagging strategy for simultaneous isolation of astrocyte, neuronal, and OPC-enriched populations from human temporal lobe neocortex. **(a) **Representative FANS pseudocolor plots showing sequential gating of non-debris (red), non-doublets (green and blue), live DAPI+ nuclei (purple), NEUN+ neuronal (red), PAX6+( NEUN–) astrocyte (orange), OLIG2+( NEUN–) OPC-enriched (green), and OLIG2^LOW^ (purple) mature oligodendroglial-enriched nuclei populations from postmortem TL control fresh-frozen neocortex. TN = triple negative (PAX6–OLIG2– NEUN–) population (black). **(b)** FANS pseudocolor plots showing alternative astrocyte isolation strategy of astrocytes from TL neocortex using SOX9 instead of PAX6, in combination with NEUN and OLIG2. **(c)** Gene expression by qRT-PCR confirms high expression of the genes used as markers for isolation and the enriched expression of the astrocytic markers *GFAP* and *ALDH1L1* in PAX6+ nuclei, the OPC markers *NG2 (CSPG4)* and *PDGFRA* in OLIG2+ nuclei, the microglial marker *CD11b* in the triple negative (TN) NeuN–PAX6–OLIG2–population, and the myelinating oligodendrocyte marker *PLP1* in the OLIG2^LOW^ region of TN. **(d)** Expression of *GFAP* and *CD44* assessed by RT-qPCR in SOX9+, PAX6+ and other FANS populations derived from TL tissue. **(e) **Representative immunofluorescence images of NEUN, OLIG2 and GFAP expression in TL control tissue. Scale bar = 50 µM.

**Additional file 2: Table S1**. Normalized RNA-seq gene expression data for all sorted nuclei, log-transformed (rld-normalized). A=Autopsy, TL; E=Epilepsy, TLE; N=NEUN+ FANS; O=OLIG2+ FANS; P=PAX6+ FANS.

**Additional file 3: Table S2**. Differential expression, TLE vs. TL, nuclear RNA-seq datasets for astrocyte, OPC, and neuronal FANS populations, with annotation of dysregulated genes unique to each cell type.

**Additional file 4: Table S3**. Functional enrichment analyses of differentially expressed genes for each nuclei cell type, TLE vs. TL, generated using DAVID.

**Additional file 5: Table S4**. Functional enrichment analyses of differentially expressed genes for each nuclei cell type, TLE vs. TL, generated using HOMER.

**Additional file 6: Table S5**. Sequencing and mapping quality control metrics of single cell RNA-seq data from five independent TLE samples.

**Additional file 7: Fig. S2**. Single cell transcriptomic analysis of integrated TLE dataset **(a) **UMAP representation of integrated TLE scRNA-seq dataset, separated by patient and colored by cell type annotation, showing contribution of all patient data to all clusters. **(b)** Heatmap representation of top 10 differential markers per cluster (FindMarkers Seurat function). Clusters are labeled according to cell type annotations in Fig. 4b. **(c) **Violin plot of log-normalized gene expression for *OLIG2* across the six subclusters identified in Fig. 4b.

**Additional file 8: Table S6**. List of top 50 cluster-defining genes in Seurat for main object integrated TLE analysis in Fig. 4a, subclustered glia object TLE analysis in Fig. 4b, and main object TL+TLE integrated analysis in Fig. 5a.

**Additional file 9: Fig. S3**. Annotation markers used in the TLE single cell transcriptomic analysis **(a-e)** Feature plots showing log-normalized expression of canonical markers used for annotation of cell types in the subclustered TLE glia analysis in Fig. 4b. (a) Astrocyte, (b) Reactive astrocyte, (c) OPC, (d) Microglia, and (e) Oligodendrocyte. **(f)** Feature plots showing log-normalized expression of canonical microglia and macrophage markers in the main integrated TLE analysis in Fig. 4a.

**Additional file 10: Fig. S4**. Single cell transcriptomic analysis of TLE + normal TL integrated dataset **(a)** UMAP representation of TLE samples integrated with TL normal data [39]. The clusters are colored and labeled according to their respective annotated cell type identity. **(b) **Feature plots showing log-normalized expression of canonical markers used for annotation of cell types in the TLE + normal TL integrated analysis from (a). **(c)** Violin plot (left) and scaled gradient feature plot (right) representing projections of normal “TL oligodendrocyte” module score signature onto the diseased TLE subclustered dataset in Fig. 4b (* = p-adj. < 0.05; ** = p-adj. < 0.005; *** = p-adj. < 0.0005 using Wilcoxon rank test, with Benjamini Hochberg correction). **(d) **UMAP representation of Monocle3 pseudotime lineage trajectory analysis of TLE subclustered glia shown with OPC (left) or Reactive astrocyte (right) as the root cluster.

## Data Availability

The data that support the findings in this study are publicly available at https://data.mendeley.com/drafts/w4d7sdc629, and are currently being deposited in NCBI's Gene Expression Omnibus to be accessible through a GEO Series accession number GSE140393 prior to publication.
