# Correction: High-resolution transcriptomics informs glial pathology in human temporal lobe epilepsy

**DOI:** 10.1186/s40478-022-01479-5

**Published:** 2022-11-28

**Authors:** Balagopal Pai, Jessica Tome‑Garcia, Wan Sze Cheng, German Nudelman, Kristin G. Beaumont, Saadi Ghatan, Fedor Panov, Elodia Caballero, Kwadwo Sarpong, Lara Marcuse, Jiyeoun Yoo, Yan Jiang, Anne Schaefer, Schahram Akbarian, Robert Sebra, Dalila Pinto, Elena Zaslavsky, Nadejda M. Tsankova

**Affiliations:** 1 Department of Pathology and Laboratory Medicine, Icahn School of Medicine at Mount Sinai, New York, NY 10029 USA; 2 Department of Neuroscience and Friedman Brain Institute, Icahn School of Medicine at Mount Sinai, New York, NY 10029 USA; 3 Department of Neurology, Icahn School of Medicine at Mount Sinai, New York, NY 10029 USA; 4 Department of Genetics & Genomic Sciences, Icahn School of Medicine at Mount Sinai, New York, NY 10029 USA; 5 Icahn Institute for Data Science and Genomic Technology, New York, NY 10029 USA; 6 Department of Neurosurgery, Icahn School of Medicine at Mount Sinai, New York, NY 10029 USA; 7 Department of Psychiatry, Icahn School of Medicine at Mount Sinai, New York, NY 10029 USA

**Correction to**: **Acta Neuropathologica Communications** **(2022) 10:149** 10.1186/s40478-022-01453-1

Following publication of the original article [1], the authors identified an error in Table 1 due to a typesetting mistake. The correctly formatted table is given below and the original article has been corrected.

**Table 1:** Sample information

Sample ID	Tissue, location	Age, Gender PMT	Nuclei RNA-SEQ	SC RNA-SEQ	Pathological diagnosis
**Cell-type specific FANS Validation, TL vs. TLE**					
10,662	TL control, temporal lobe neocortex	34yo, M 12 h PMT	✓		No neuropathological changes seen in cortex
10,355	TL control, temporal lobe neocortex	45yo, F 12 h PMT	✓		No neuropathological changes seen in cortex
10,997	TL control, temporal lobe neocortex	27yo, M 21 h PMT	✓		No neuropathological changes seen in cortex
12,321	TLE, temporal lobe neocortex	50yo, M	✓		Cortical and Chaslin Gliosis; White matter neuronal heterotopia
10,308	TLE, temporal lobe neocortex	29yo, F	✓		Cortical and Chaslin gliosis; FCD Ia
12,726	TLE, temporal lobe neocortex	13yo, M	✓		Cortex without pathologic changes; *
14,431	TLE, temporal lobe neocortex	28yo, M		✓ (10X, v2)	Cortical Gliosis; Ectopic white matter neurons with mild hypertrophy;
19,619	TLE, temporal lobe neocortex	31yo, F		✓ (10X, v2)	Chaslin gliosis; *
20,188	TLE, temporal lobe neocortex	58yo, F		✓ (10X, v3)	Cortical and Chaslin gliosis, rare neurons with hypertrophy and disoriented dendrites; *
12,814	TLE, temporal lobe neocortex	11yo, M		(10X, v1)	Chaslin gliosis; *
13,059	TLE, temporal lobe neocortex	8yo, M		✓ (10X, v1)	Gliosis, neuronal heterotopia;

Sample ID	Tissue, location	Age, Gender	Ki-67 IF	In vitro assays	Pathological diagnosis
**Phenotypic analysis of epilepsy glia**					
12,321	TLE, temporal lobe neocortex	50yo, M	✓’ (high)	✓	Cortical and Chaslin Gliosis; White matter neuronal heterotopia;
10,308	TLE, temporal lobe neocortex	29yo, F	✓(low)	✓	Cortical and Chaslin gliosis; FCD Ia;
12,726	TLE, temporal lobe neocortex	13yo, M	✓’ (high)		Cortex without pathologic changes; *
14,431	TLE, temporal lobe neocortex	28yo, M	✓(low)	✓	Cortical Gliosis; Ectopic white matter neurons with mild hypertrophy;
12,319	Epilepsy, frontal lobe neocortex	27yo, M	✓’ (high)		Chaslin Gliosis; Ischemic changes
12,433	Epilepsy, TS, frontal lobe neocortex	4yo, M	✓(low)	✓	Cortical and Chaslin Gliosis; **

*PMT* Postmortem time, *IF* Immunofluorescence, *M* Male, *F* Female, *yo* years old; *h* hours; *TS* Tuberous sclerosis. *FCD* Focal cortical dysplasia

^*^Hippocampal sclerosis seen away from the sampled area, ^**^Tubers seen away from the sampled area.’ Samples included in the IF analysis of Ki67 proliferation cell type distribution

In addition, the authors corrected the ‘Availability of data and materials’ section.

The statement in the ‘Availability of data and materials’ section originally read: The data that support the findings in this study are publicly available at https://data.mendeley.com/drafts/w4d7sdc629, and are currently being deposited in NCBI’s Gene Expression Omnibus to be accessible through a GEO Series accession number GSE140393 prior to publication.

The statement in the ‘Availability of data and materials’ section should read: The data that support the findings in this study are publicly available in NCBI’s Gene Expression Omnibus through a GEO Series accession number GSE140393.
