# Supplementary figures and images for: High-resolution transcriptomics informs glial pathology in human temporal lobe epilepsy
Source: Acta Neuropathol Commun. 2022 Oct 23;10:149. doi: 10.1186/s40478-022-01453-1 (PMC9590125; doi:10.1186/s40478-022-01453-1)

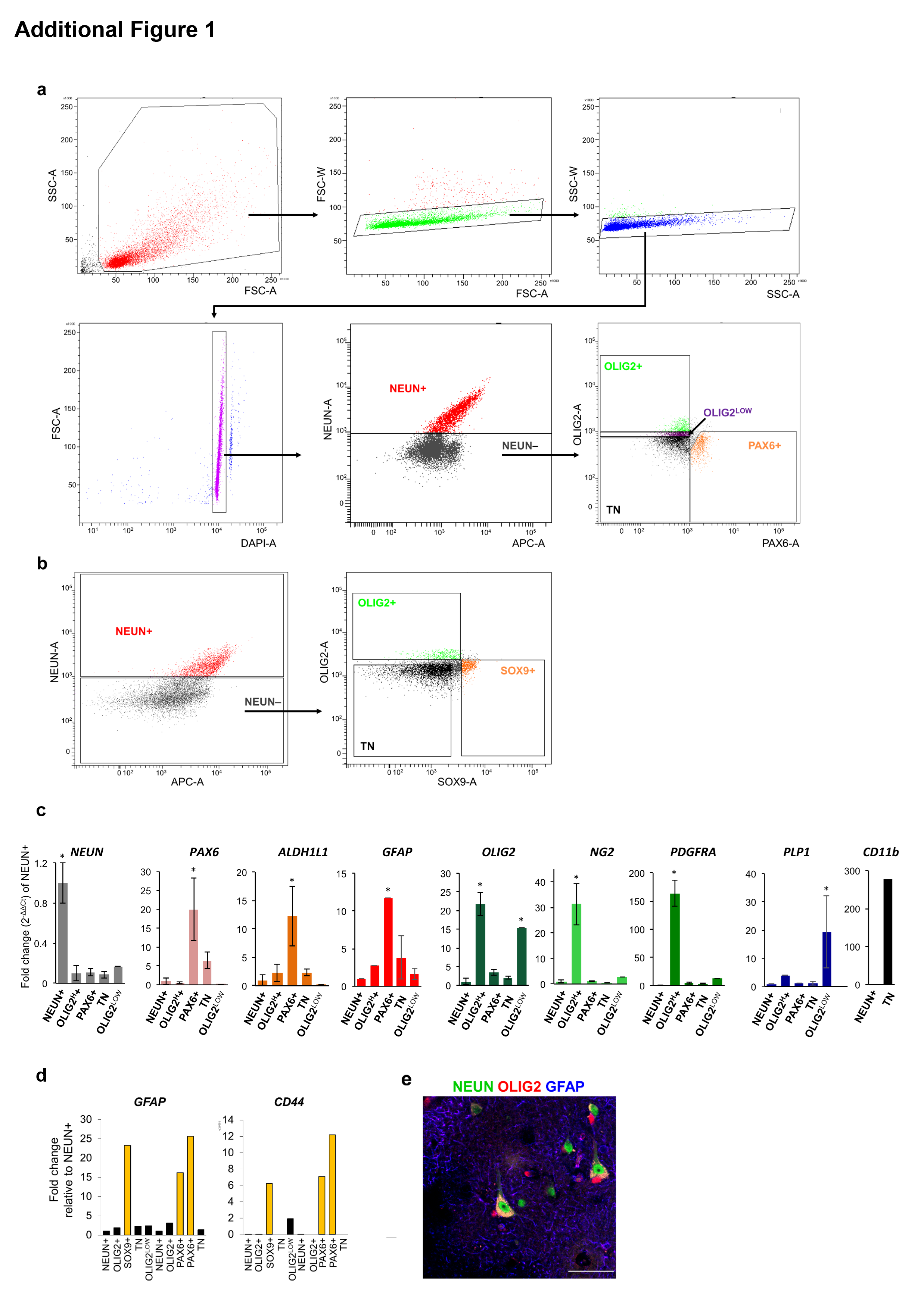

Supplement: Supplementary file 1 — Additional file 1: Fig. S1. Immunotagging strategy for simultaneous isolation of astrocyte, neuronal, and OPC-enriched populations from human temporal lobe neocortex. (a) Representative FANS pseudocolor plots showing sequential gating of non-debris (red), non-doublets (green and blue), live DAPI+ nuclei (purple), NEUN+ neuronal (red), PAX6+( NEUN–) astrocyte (orange), OLIG2+( NEUN–) OPC-enriched (green), and OLIG2LOW (purple) mature oligodendroglial-enriched nuclei populations from postmortem TL control fresh-frozen neocortex. TN = triple negative (PAX6–OLIG2– NEUN–) population (black). (b) FANS pseudocolor plots showing alternative astrocyte isolation strategy of astrocytes from TL neocortex using SOX9 instead of PAX6, in combination with NEUN and OLIG2. (c) Gene expression by qRT-PCR confirms high expression of the genes used as markers for isolation and the enriched expression of the astrocytic markers GFAP and ALDH1L1 in PAX6+ nuclei, the OPC markers NG2 (CSPG4) and PDGFRA in OLIG2+ nuclei, the microglial marker CD11b in the triple negative (TN) NeuN–PAX6–OLIG2–population, and the myelinating oligodendrocyte marker PLP1 in the OLIG2LOW region of TN. (d) Expression of GFAP and CD44 assessed by RT-qPCR in SOX9+, PAX6+ and other FANS populations derived from TL tissue. (e) Representative immunofluorescence images of NEUN, OLIG2 and GFAP expression in TL control tissue. Scale bar = 50 µM. [file 40478_2022_1453_MOESM1_ESM.tif]

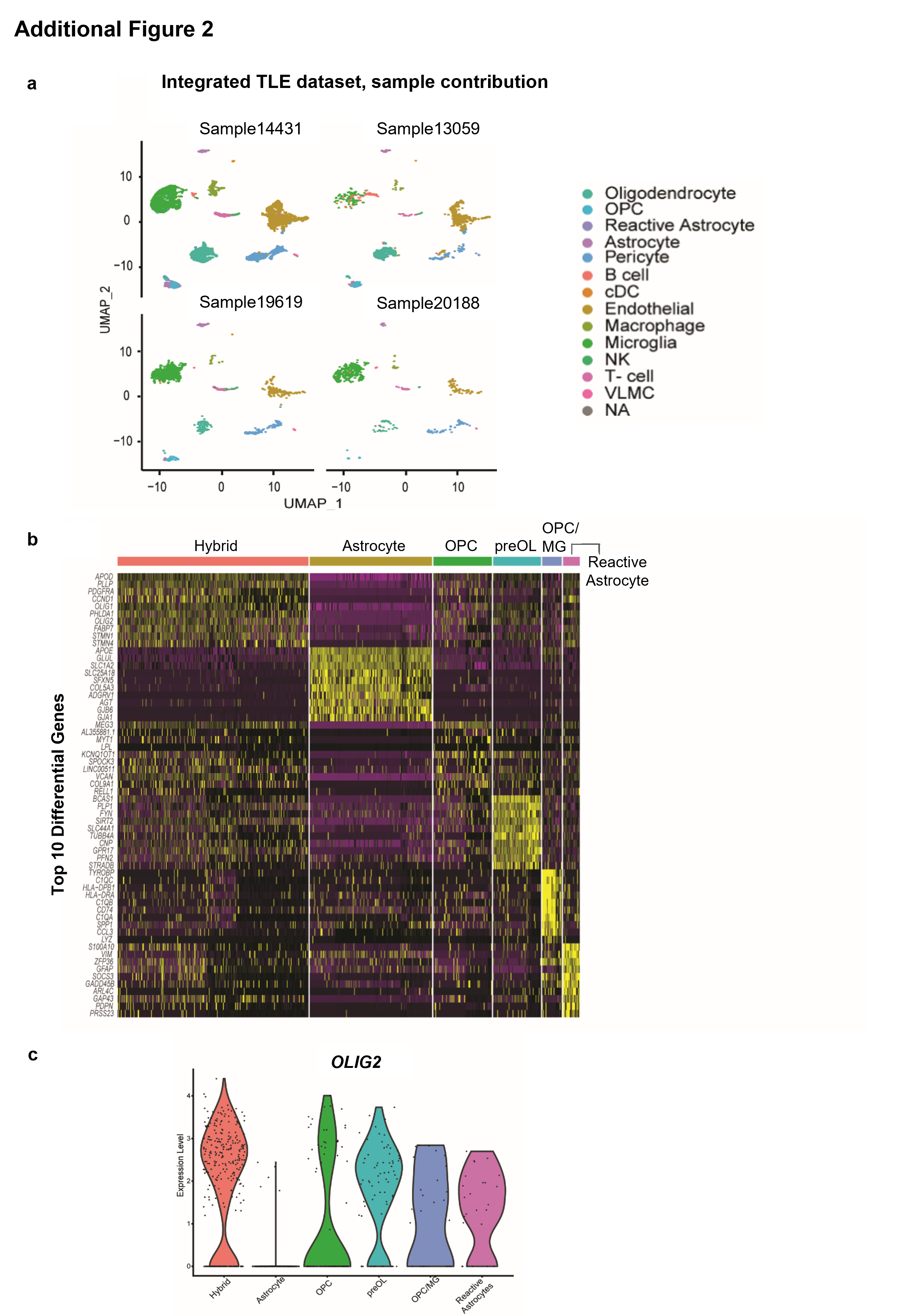

Supplement: Supplementary file 7 — Additional file 7: Fig. S2. Single cell transcriptomic analysis of integrated TLE dataset (a) UMAP representation of integrated TLE scRNA-seq dataset, separated by patient and colored by cell type annotation, showing contribution of all patient data to all clusters. (b) Heatmap representation of top 10 differential markers per cluster (FindMarkers Seurat function). Clusters are labeled according to cell type annotations in Fig. 4b. (c) Violin plot of log-normalized gene expression for OLIG2 across the six subclusters identified in Fig. 4b. [file 40478_2022_1453_MOESM7_ESM.tif]

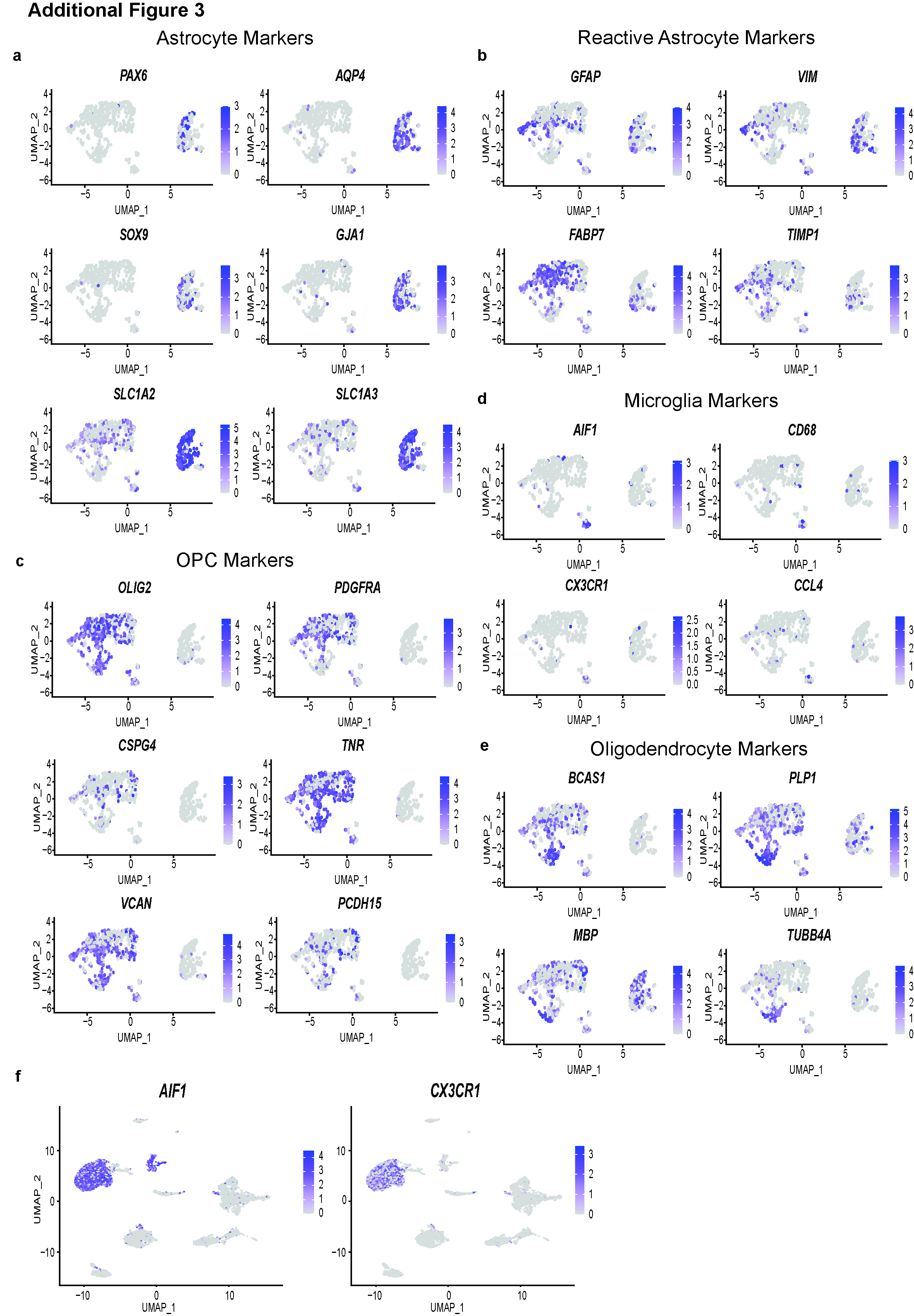

Supplement: Supplementary file 9 — Additional file 9: Fig. S3. Annotation markers used in the TLE single cell transcriptomic analysis (a-e) Feature plots showing log-normalized expression of canonical markers used for annotation of cell types in the subclustered TLE glia analysis in Fig. 4b. (a) Astrocyte, (b) Reactive astrocyte, (c) OPC, (d) Microglia, and (e) Oligodendrocyte. (f) Feature plots showing log-normalized expression of canonical microglia and macrophage markers in the main integrated TLE analysis in Fig. 4a. [file 40478_2022_1453_MOESM9_ESM.tif]

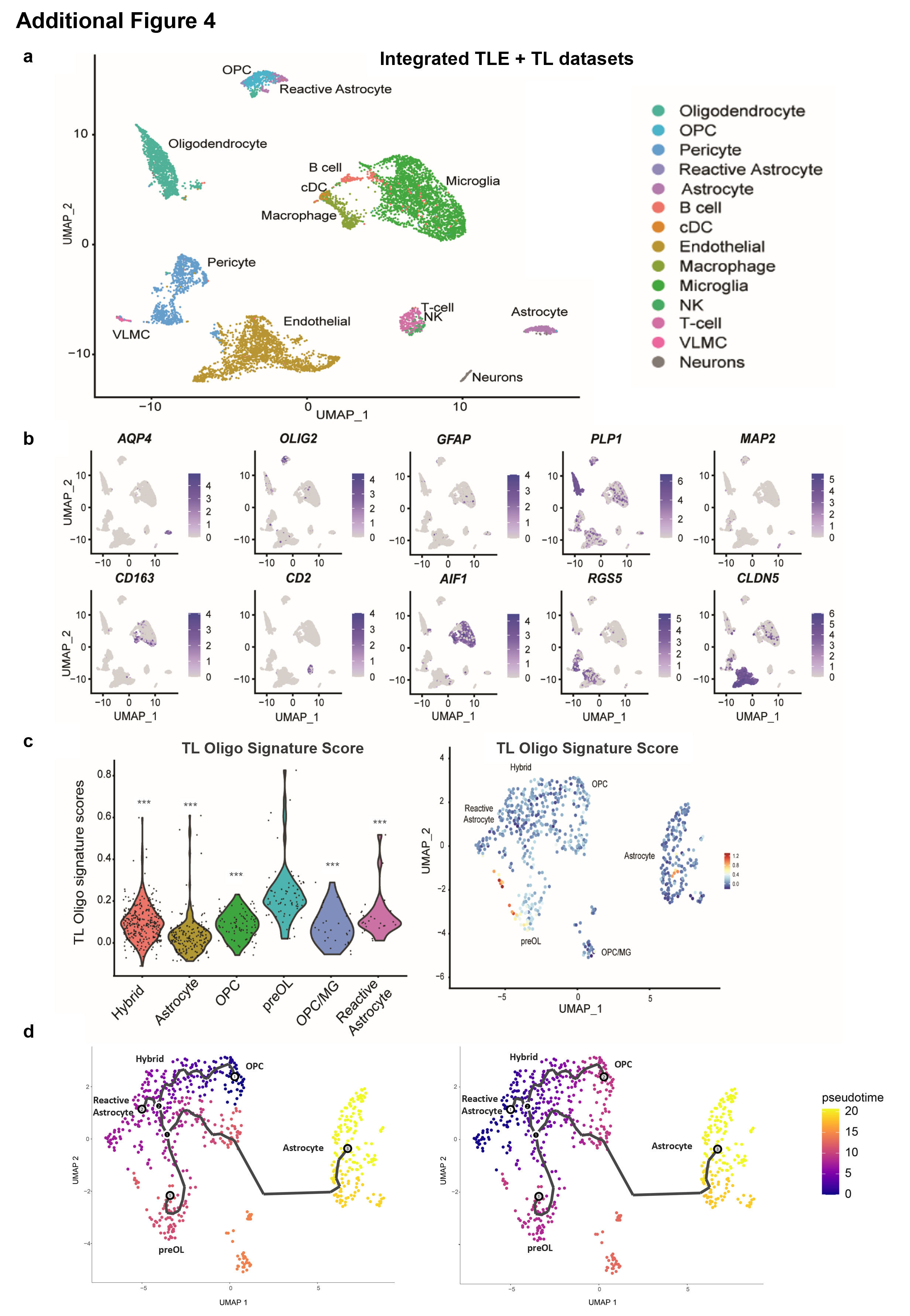

Supplement: Supplementary file 10 — Additional file 10: Fig. S4. Single cell transcriptomic analysis of TLE + normal TL integrated dataset (a) UMAP representation of TLE samples integrated with TL normal data [39]. The clusters are colored and labeled according to their respective annotated cell type identity. (b) Feature plots showing log-normalized expression of canonical markers used for annotation of cell types in the TLE + normal TL integrated analysis from (a). (c) Violin plot (left) and scaled gradient feature plot (right) representing projections of normal “TL oligodendrocyte” module score signature onto the diseased TLE subclustered dataset in Fig. 4b (* = p-adj. < 0.05; ** = p-adj. < 0.005; *** = p-adj. < 0.0005 using Wilcoxon rank test, with Benjamini Hochberg correction). (d) UMAP representation of Monocle3 pseudotime lineage trajectory analysis of TLE subclustered glia shown with OPC (left) or Reactive astrocyte (right) as the root cluster. [file 40478_2022_1453_MOESM10_ESM.tif]
